# Multiplexed, rapid phenotypic antibiotic susceptibility testing based on angle-resolved light scattering imaging of microfluidic droplets

**DOI:** 10.1016/j.jare.2025.09.047

**Published:** 2025-09-25

**Authors:** Martina Graf, Arjun Sarkar, Carl-Magnus Svensson, Anne-Sophie Munser, Sven Schröder, Elke Müller, Sundar Hengoju, Marc Thilo Figge, Miriam A. Rosenbaum

**Affiliations:** a Bio Pilot Plant, Leibniz Institute for Natural Product Research and Infection Biology – Hans-Knöll-Institute HKI, 07745 Jena, Germany; b Institute of Microbiology, Faculty of Biological Sciences, Friedrich Schiller University, 07745 Jena, Germany; c Applied System Biology, Leibniz Institute for Natural Product Research and Infection Biology – Hans-Knöll-Institute HKI, 07745 Jena, Germany; d Functional Surfaces and Coatings, Fraunhofer Institute for Applied Optics and Precision Engineering IOF, 07745 Jena, Germany; e Optical Molecular Diagnostics and System Technology, Leibniz Institute of Photonic Technology, 07745 Jena, Germany; f InfectoGnostics Research Campus Jena, Center for Applied Research, 07745 Jena, Germany; g Cluster of Excellence Balance of the Microverse, Friedrich Schiller University, 07745 Jena, Germany

**Keywords:** Rapid antibiotic susceptibility testing, Angle-resolved light scattering imaging, Droplet microfluidics, Convolutional neural networks, *Staphylococcus aureus*

## Abstract

**Introduction:**

There is an urgent need for rapid, high-throughput phenotypic antimicrobial susceptibility testing (AST) capable of assessing a microbial sample’s susceptibility to multiple antibiotics.

**Objectives:**

In this study, we have established a multiplexed rapid AST platform that employs droplet microfluidics for high-throughput single-cell based analysis, 2D angle-resolved light scattering for growth detection, and fluorescence detection via optical fibers to identify the antibiotic condition within each droplet.

**Methods:**

For this, multiple antibiotic conditions are coded with fluorescence dyes and encapsulated with single cells to enable the testing of multiple antibiotics in a single experiment. We utilize convolutional neural networks (CNNs) and statistical models to assess the growth of various *Staphylococcus aureus* strains and determine the probability of susceptibility to different antibiotics.

**Results:**

Our platform achieved a 95 % categorical agreement with the disc diffusion reference method after just three hours of incubation, demonstrating the same level of accuracy as the established VITEK 2 system for the tested strains and antibiotics. Notably, our platform reduced the incubation time by 5–11 h compared to VITEK 2 and by 13–17 h compared to the gold standard disc diffusion method.

**Conclusions:**

With the presented innovations, our technology takes a big step towards realizing true phenotypic determination of antibiotic resistance profiles for timely antimicrobial treatment decisions.

## Highlights

• Rapid antimicrobial susceptibility testing via 2D light scattering, fluorescence detection and droplet microfluidics.
• Tens of thousands of picoliter droplets are assessed for both cell concentration and antibiotic conditions.
• A resistance/susceptibility profile is obtained within just three hours of incubation.
• This is a substantial improvement over conventional phenotypic AST methods.
• The platform is reliable and robust, as demonstrated by a 95% categorical agreement with the reference disc diffusion method.

## Graphical abstract

**Figure:**
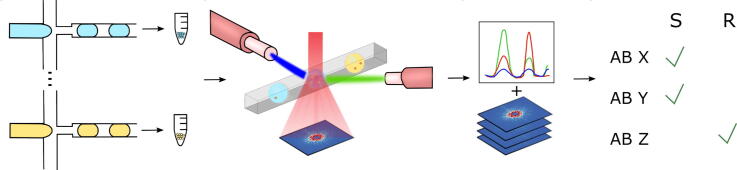

## Introduction

The rise of multi-drug-resistant bacteria threatens to bring about a “post-antibiotic era”, where infections that were once easily treated become deadly [1,2]. Without effective measures, these resistant infections could cause around 10 million deaths per year by 2050, surpassing the death toll from cancer [3]. The rapid increase of antibiotic-resistant bacteria is driven by the misuse of antibiotics, such as using incorrect antibiotics or administering the right ones at improper doses. This misuse promotes the development of resistant bacteria through natural selection. The World Health Organization (WHO) has identified antibiotic resistance as one of the top ten global public health threats [4]. The slow pace of new antibiotic development makes it crucial to find faster ways to test bacteria for antibiotic susceptibility [5,6]. Rapid diagnostic tools are essential to reduce the use of broad-spectrum antibiotics, support targeted treatments, and monitor antibiotic resistance. This is especially important for fast growing pathogens with a high potential for antibiotic resistances like *Staphylococcus aureus*, where every hour might count to reduce the infection impact on the patient.

Current gold standards for antimicrobial susceptibility testing (AST) are based on growth detection such as disk diffusion [7] and broth microdilution [8]. There are also more automated approaches such as VITEK® 2 (bioMérieux) and the Phoenix^TM^ automated microbiology system (BD Diagnostics). These methods are reliable and widely used in clinical settings; however, they are inherently time-consuming, often requiring 8 to 20 h of incubation to yield results [9]. In the context of severe infections, this delay can be harmful, as it postpones the administration of effective targeted therapies, potentially worsening patient outcomes. Besides phenotypic ASTs, genotypic ASTs have been developed to rapidly detect drug resistance genes in bacterial pathogens. The advantage of them are, that they are not dependent on growth and can therefore deliver results in a very short time, sometimes within an hour [10]. Some techniques combine genotypic resistance detection with pathogen identification, such as detecting the mecA gene for methicillin-resistant *S. aureus* (MRSA) [11]. However, the absence of a resistance gene does not always indicate susceptibility, as other resistance mechanisms like porin loss and efflux pumps can be present [12]. Thus, genotypic testing supplements rather than replaces phenotypic susceptibility testing, helping to exclude certain antibiotics for treatment by identifying specific resistance genes. Consequently, there is an urgent need for new methods of rapid phenotypic AST.

Droplet-based microfluidics presents a promising alternative to conventional low-throughput antibiotic resistance tests. This technology involves creating tiny droplets of usually water in oil in high-throughput [13,14], allowing millions of tests to be conducted simultaneously [15,16]. Droplet microfluidics enables handling of liquids on a much smaller scale, ranging from femtoliters to nanoliters, speeding up the testing process. Each droplet can contain different reagents and/or cells, making it possible to test many conditions in parallel. If multiple conditions are generated, then barcoding the droplets is necessary, so the experimental results can be matched to the corresponding experimental condition. Spatial barcoding of the droplets is possible by separating the droplet conditions through an immiscible phase [17,18]. Identification is also possible with oligonucleotide barcodes [19,20], colored beads [21] or by color coding the droplets with one or more dyes, either fluorescence [22,23] or food dye [24].

In droplet-based phenotypic AST, single cells are usually encapsulated to classify the level of resistance, measure the minimal inhibitory concentration, or both [21,[25], [26], [27]]. The advantage of droplets is that rare resistant-variant subpopulations that might be under the detection limit with classical methods can be detected [28]. Therefore, some bacteria subpopulations might be misclassified with standard diagnostic methods, resulting in treatment failure [29,30].

The early detection of microbial growth from single cells plays a crucial role for rapid growth-based AST. Several detection strategies have been utilized such as the use of various biosensors, for example for the detection of oxygen consumption or the pH of the droplet environment as an indirect measurement of growth [31,32]. More direct measuring techniques involve measuring fluorescence [25] or scattered light at a single angle [33,34] or 2D angle-resolved [[35], [36], [37], [38]].

In 2D angle-resolved light scattering (ARS) imaging, cells are illuminated by a laser and the scattered light of that laser is captured at a broad range of angles by a detector matrix. We have shown in our previous work, that 2D ARS imaging is a very sensitive technique that enables bacterial growth detection from single cells in droplets in flow after only a single hour of incubation for *E. coli* and after two hours for *S. aureus*. Additionally, we demonstrated in a proof-of-principle study that antibiotic susceptibility could be determined after two hours of incubation [38]. In this work, we utilize the previously established 2D ARS imaging platform and introduce a multiplexing experimental setup in which the susceptibility of fast growing *S. aureus* strains towards multiple antibiotics can be tested on a single-cell level within a single experiment. Through fluorescence coding, the antibiotic condition can be matched to the growth behavior determined by convolutional neural network (CNN) based regression models that analyze the ARS images. This setup allows for the analysis of tens of thousands of droplets with various antibiotic conditions, significantly increasing throughput, reducing the time required to obtain a resistance profile, and providing more detailed insights into bacterial resistance compared to traditional bulk experiments.

## Methods

### ARS imaging setup

The same ARS sensor as described in Graf and Sarkar *et al.* [38] has been used with some changes and additions [35,39]. The illumination path consists of a 660  nm diode laser source, beam preparation optics including spatial filter for a clean Gaussian beam, and a focusing lens that generates a spot size of 25  µm (at e^-2^ intensity) with approximately 0.5 mW power in the droplet plane with an incidence angle θ_i_ = 0°. A CMOS detector matrix (VLXT-71 M.I, Baumer) is utilized for 2D angular scattering data acquisition in a single shot. The specular transmitted beam with an extension of |θ_s_|= 4° on the matrix of the detector is blocked by a beam dump. The maximum detectable polar scattering angles are |θ_s,d_| = 37° for the diagonal and |θ_s,h_| = 30° and |θ_s,v_| = 20° for the horizontal and vertical sides, respectively. The detectable azimuthal angles are ϕ_s_ = ±180°. The angles are determined by the geometric dimensions and distance of the CMOS detector. A bandpass filter was placed on top of the detector matrix to block the fluorescence signal from the fluorescence dyes. Triggered ARS images were acquired with 400  µs and 800  µs exposure times. An optical microscope (12x magnification) was used to monitor the imaging position.

### Fluorescence detection setup

The fluorescence was detected as described by Tovar *et al*. [40]. In short, the core and cladding layer of the optical fiber were exposed at the tips and were cleaved with a fiber cleaver to create a flat surface. The optical fiber tips were inserted into the fiber guiding structures of the microfluidic chip and fixed with instant glue to the glass slide. Two multimode fibers (FC/PC fiber patch cable, 0.22 numerical aperture, 105 + 1/-3 µm core diameter, Thorlabs) were used: one for excitation and one for detection. The excitation fiber was connected to a beam combiner consisting of three modulated lasers with different wavelengths (408  nm, 488  nm, 561  nm). The detection fiber was connected to a photomultiplier tube with a quad-bandpass filter (440/521/607/700 HC quadband filter, AHF). A lock-in amplifier (HF2LI, Zurich Instruments) filtered signals according to each laser modulation frequency, creating three independent outputs. The fluorescence outputs were recorded and visualized using a DAQ card (USB-1608GX, Measurement Computing) and a custom-written LabView program. Further processing and analysis of the fluorescence data and the matching of the fluorescence data to the ARS images was performed using python.

For the identification of the experimental condition based on fluorescence, the heights and widths of the fluorescence signals were analyzed. Satellite and merged droplets were filtered out based on the blue fluorescence signals. The green and red fluorescence signals were normalized by the blue fluorescence and split into three groups: containing only red, only green or both/neither fluorescence. GaussianMixture was used to cluster the groups containing only red and only green based on the normalized signals. Afterwards, all groups were clustered with DBSCAN to remove noise.

### Microorganisms and culture conditions

*S. aureus* ST033804 from the Jena Microbial Resource Collection was used in the dilution series for training the CNN. Four *S. aureus* strains, provided by the Leibniz-IPHT, were used for the AST experiments (Table 1). Pre-cultures were made from cryo-stocks and incubated at 37 °C overnight. Main cultures were prepared with a starting optical density (OD) at 600 nm of 0.1 (BioPhotometer, Eppendorf) and incubated until they reached an OD between 1 and 2. Then, ODs were set depending on the experiments conducted. Lysogeny broth (LB) was used as medium. LB media was prepared by mixing 10 g/L tryptone (Bacto Tryptone, BD Bioscience), 5 g/L yeast extract (Bacto Yeast Extract, BD Bioscience), and 10 g/L NaCl (Merck, Germany) in tap water, pH adjusted to 7 with NaOH, and autoclaving for 20 min at 121 °C.

**Table 1:** List of *S. aureus* strains used in the AST experiments. R = resistant, I = susceptible with increased exposure, S = susceptible

No.	Strain designation	Resistance trait (VITEK-2)	Reference
1	CC22-MRSA-IV, Barnim“(IsolateA257)	R: PEN, GEN, ERY, CIP	BacDive ID: 160,395
		S: TET	
2	CC8-MSSA NCTC 8325	S: TET, CIP, ERY, GEN, PEN	GenBank CP000253.1
			BacDive ID: 14,451
3	caMRSA, CC80-MRSA-IV with PVL	R: TET, PEN	[41]
		I: CIP	
		S: GEN, ERY	
4	USA300_TCH1516	R: ERY, PEN	GenBank CP000730.1
		I: CIP	
		S: TET, GEN	

**Fig. 4:**
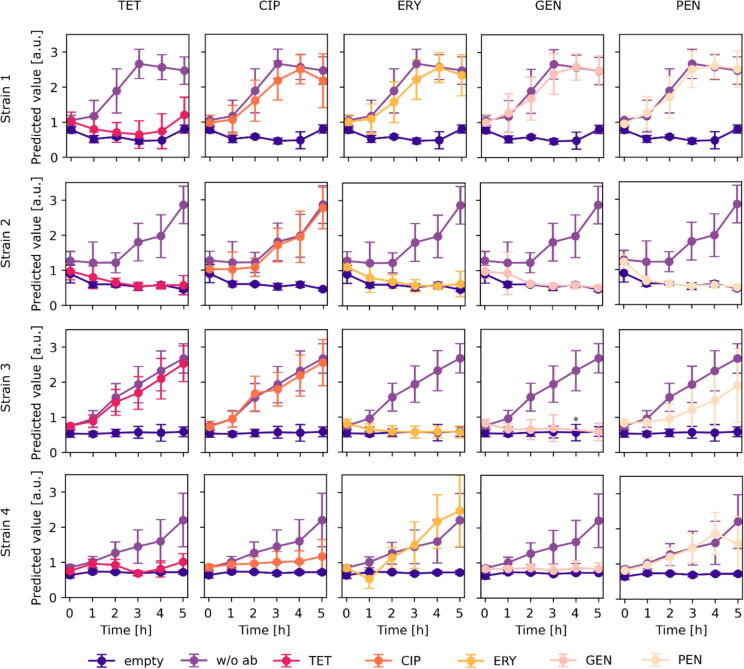
Prediction results of rapid multiplexed AST with four different *S. aureus* strains and seven conditions (1  µg/mL TET, 0.001  µg/mL CIP, 1  µg/mL ERY, 2  µg/mL GEN, 0.125  µg/mL PEN, without antibiotic, no cells). Droplets were incubated and characterized with the ARS and fluorescence platform hourly. The predicted values of each antibiotic condition are displayed compared to the two control populations (empty droplets and droplets with cells but no antibiotic = uninhibited). ★ = no ARS images were matched to this condition at 4  h.

**Fig. 5:**
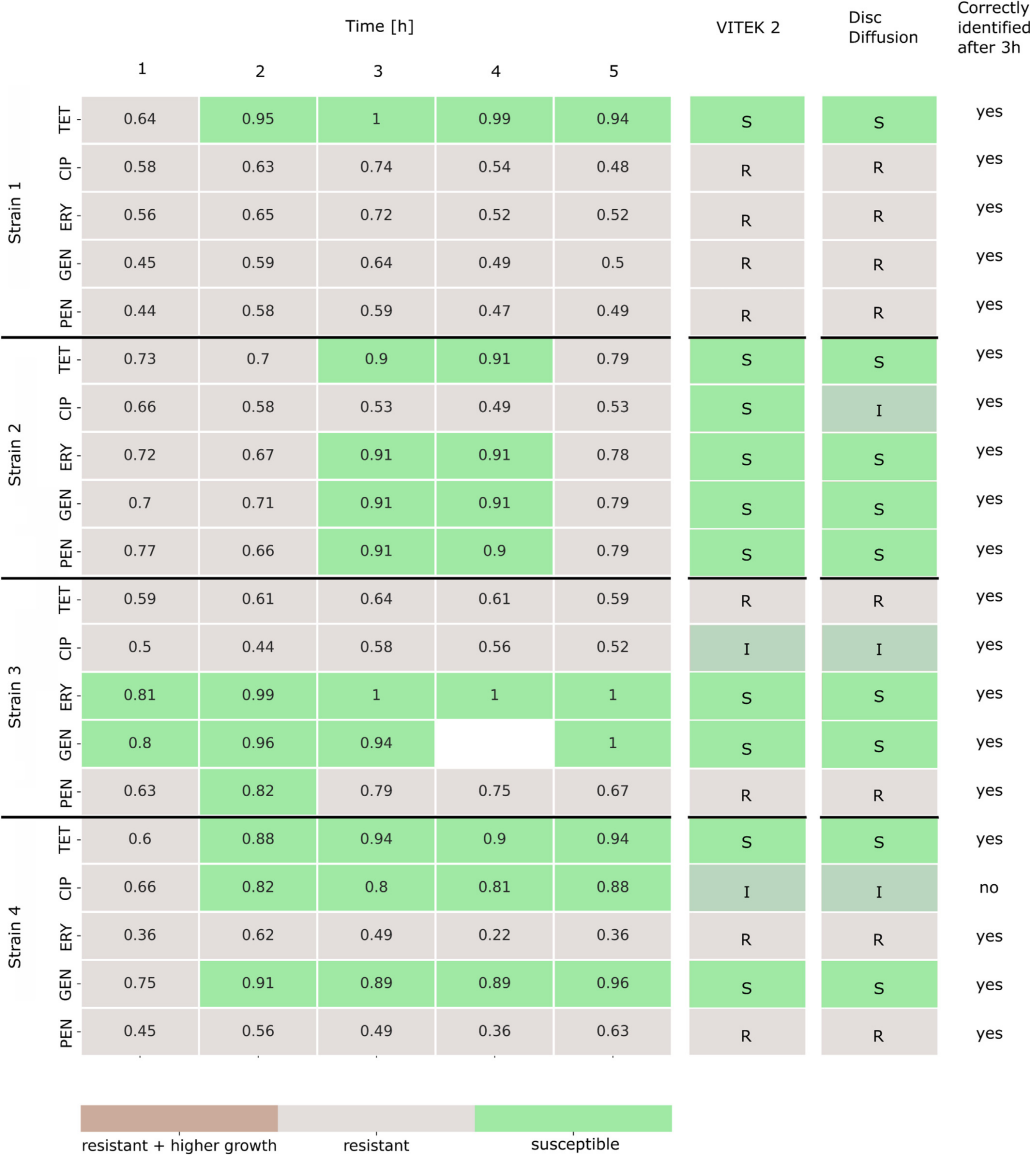
Probabilities that the tested organisms are susceptible or resistant to each tested antibiotic at each timepoint. Antibiotic breakpoint concentrations described by EUCAST were used. The lower breakpoint concentration was used for CIP. Probability values >= 0.8 are considered as susceptible. Values below 0.8 indicate resistant strains, while below 0.2 the antibiotic condition caused an increase in growth in comparison to the absence of an antibiotic. The probability results are compared to results derived from VITEK 2 and disc diffusion assay.

The different droplet conditions, i.e. antibiotic type and concentration plus a corresponding fluorescence code, were assembled manually before droplet generation. 20 or 40  µL/mL of 4  mg/mL green fluorescent dye (Dextran, Alexa Fluor^TM^ 488, Invitrogen^TM^) in DMSO and/or 4, 8 or 12  µL/mL of 1  mg/mL red fluorescent dye (DY-557, carboxylic acid, Dyomics) in DMSO was added for coding the experimental conditions. 5 µL/mL of 1 mg/mL blue fluorescent dye (DY-405, carboxylic acid, Dyomics) in DMSO was added as a general droplet label for triggering the analytical setup. Antibiotics were added to the droplets before droplet generation as well for experiments displayed in Figs. 4 and 5. Antibiotic concentrations of 1 µg/mL tetracycline (TET, Sigma), 0.001  µg/mL ciprofloxacin (CIP, AppliChem), 1  µg/mL erythromycin (ERY, Roth), 2  µg/mL gentamycin (GEN, Roth) and 0.125  µg/mL benzylpenicillin (PEN, Fluka) were used for AST.

### Microfluidic mold and chip fabrication

Microfluidic chips for droplet generation and ARS imaging/fluorescence detection were designed using AutoCAD (Autodesk Corp.) and manufactured in-house. The SU8-2050 photoresist mold was fabricated using photolithography following the manufacturer’s protocol (Microchem). Two layers with heights of 50  µm (microfluidic structures) and 75  µm (fiber guiding and lens structures) were fabricated onto a silicon wafer with SU8-using photomasks and UV exposure (Kloe UV3). For the first layer, the SU8 on the wafer was exposed for 10  s and the second for 15  s, both with 20 % lamp power. The fabrication process for the femtoprinted chips is explained in detail by Tovar *et al.* as well as the polydimethylsiloxane (PDMS) soft lithography that was utilized to form microfluidic chips from the wafers [42].

### Droplet generation and incubation

An OB-1 pressure pump (Elveflow) was used to guide the liquids through polytetrafluoroethylene (PTFE) tubings into a microfluidic chip and to a flow-focusing structure with a nozzle size of 50  µm. Droplets were generated with Novec HFE7500 oil with 0.4 % (v/v) FluoSurf (Emulseo). The generated droplets (∼55–60  µm diameter) were collected through PTFE tubings in microreaction vessels. They were either directly transferred to the imaging and detection platform or incubated at 37 °C for 1–5  h.

### Microscopic imaging

Stationary droplets were imaged in an observation chamber with a PCO.edge 5.5  m camera (PCO) and a Spectra-X Light Engine (Lumencor) using an inverted microscope (Axio Observer Z1, Carl Zeiss).

### Deep learning analysis

Analysis was conducted as described by Graf and Sarkar *et al.* [38]. In short, a supervised deep learning model, EfficientNetB4 [43], was trained to identify and remove falsely triggered images (Section S3.2). The trained EfficientNetB4 model was subsequently applied to all ARS images, to filter out falsely triggered images. After this filtering process, a total of 54,000 images remained for training, 3,000 images for validation, and 44,983 images for testing a CNN-based regression model aimed at predicting values as a measure of cell concentration. For this regression task, a pretrained EfficientNetV2-XL model [44], initialized with ImageNet21K weights [45], was employed as the CNN architecture (Section S3.2).

### Statistical analysis

Each experiment contains an empty droplet population, which is used to determine the threshold for growth for each time point, t, in each experiment. We used the 99^th^ percentile to define the threshold $T_{t}$ and considered the growth distribution above this threshold.

As described by Graf and Sarkar et al. [38], we describe the growth distribution of condition c at time t by fitting the function

(1) $$p\left({\mathrm{Growth}}_{t}|c\right)=\pi_{0,t,c}+\pi_{1,t,c}lognorm\left(\mu_{t,c},\sigma_{t,c}\right)$$

where we decide $\pi_{1,t,c}$ to be the fraction of droplets with ${\mathrm{Growth}}_{t}>T_{t}$ and $\pi_{0,t,c}=1-\pi_{1,t,c}.$ The parameters $\mu_{t,c}$ and $\sigma_{t,c}$ are determined by the mean and standard deviation of the natural logarithm of the predicted value in droplets above the threshold.

To determine the susceptibility or resistance of an antibiotic, we compare the predicted value distribution of each antibiotic, AB, with the predicted value distribution of control growth, CG above the threshold. Given the formulation in Eqn. (1), the distributions above the threshold is fully described by the parameters $\mu_{t,c}$ and $\sigma_{t,c}$ from the Normal distribution. This means that we can directly calculate the probability that the control growth condition exhibits more growth than the antibiotic AB by the equation

(2) $$p\left({\mathrm{Growth}}_{\mathrm{CG}}>{\mathrm{Growth}}_{\mathrm{AB}}\right)=\frac{1}{2}\text{erfc}\left(-\frac{\mu_{\mathrm{CG}}-\mu_{\mathrm{AB}}}{\sqrt{2\left(\sigma_{\mathrm{CG}}^{2}+\sigma_{\mathrm{AB}}^{2}\right)}}\right)$$

where erfc is the complementary error function.

Bacteria are considered to be susceptible to an antibiotic if $p\left({\mathrm{Growth}}_{\mathrm{CG}}>{\mathrm{Growth}}_{\mathrm{AB}}\right)\geq 0.8$.

The full deep learning codebase and all scripts for statistical analysis are publicly available at https://github.com/applied-systems-biology/Multiplexing-Deep_learning.

### AST with disc diffusion method

Pre-cultures from the *S. aureus* strains were incubated over night at 37 °C and diluted to OD_600_ 0.1 and incubated for another three hours. Cells were diluted with saline to a McFarland standard of 0.5 and plated on agar plates. Antibiotic discs with the recommended concentrations by EUCAST were placed onto the agar plates and then plates were incubated for 20 h at 37 °C. The inhibition zone diameters were determined with a ruler.

### AST with VITEK2 system

Antimicrobial susceptibility testing was also performed using the VITEK2 system (bioMérieux Deutschland GmbH, Nürtingen, Germany) with the AST-P608 cards according to the manufacturer's instructions. Bacterial isolates were cultured overnight at 37 °C on Columbia blood agar, cells were inoculated in 0.45 % sodium chloride to reach a McFarland standard of 0.5. For resistance testing of Gram-positive bacteria, the prepared bacterial suspension was diluted in 0.45 % sodium chloride and then loaded into the VITEK 2 system for automated analysis. The results were interpreted according to the latest EUCAST breakpoints to classify isolates as susceptible, intermediate or resistant.

### Ethics statement

This work did not involve any animal or human samples. No ethical approvals were required for this work.

## Results and discussion

### Overview of developed detection platform

**Fig. 1:**
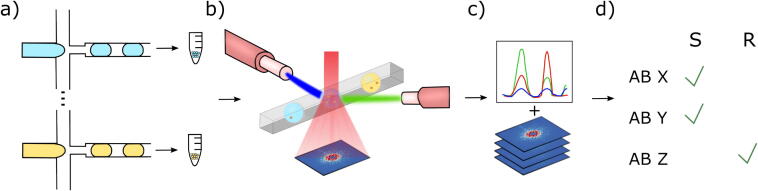
Workflow of the developed detection platform. a) Droplet populations containing different fluorescent dyes, antibiotics, and *S. aureus* cells were generated and incubated for different periods of time. b) Droplets were analyzed in flow regarding their fluorescence code via optical fibers and their cell concentrations via 2D ARS imaging. c) The fluorescence signals were analyzed and matched to the corresponding ARS image of the same droplet. ARS images were analyzed with a CNN regression model to identify the cell concentration. d) The susceptibility of the organism against the various tested antibiotics is determined.

To establish a robust multiplexed detection platform, we utilized droplet encoding based on fluorescence dyes and fluorescence signals to trigger ARS image acquisition (Fig. 1 and Section S1). The general workflow consisted of cell encapsulation into monodisperse picoliter-sized droplets with an antibiotic and fluorescence dyes (see exemplary coefficient of variant analysis of droplet size and content in Section S1). A blue fluorescent dye was added to each sample as a droplet marker while green and red fluorescent dyes were added to create a specific fluorescent code for each antibiotic. The droplet populations were collected in incubation vessels and then either incubated or directly transferred to the 2D ARS imaging and fluorescence detection platform (Fig. 1a). The droplet populations, containing different antibiotic conditions, were mixed and injected into a microfluidic chip for analysis. The fluorescently labelled droplets were excited by three lasers, guided through an optical fiber. The fluorescent signals were captured by a second optical fiber and detected by a photomultiplier tube. Droplets were detected based on the blue fluorescence signal which was used to create a trigger signal, for 2D ARS image acquisition (Fig. 1b). Fluorescence signals in the other channels (red and green) and ARS images were recorded separately. The ARS images were analyzed with a CNN and matched to the fluorescence signals. Thereby, the antibiotic condition of each imaged droplet could be determined (Fig. 1c). Finally, a decision about the susceptibility of each tested antibiotic could be made with a developed statistical model based on thousands of replicates (Fig. 1d).

### Coupling of fluorescence detection via optical fibers with ARS imaging

To couple fluorescence signals and ARS images from individual droplets, several things needed to be considered. A microfluidic chip was designed to integrate fluorescence measurements via optical fibers and ARS imaging (Section S2.1). The guiding structures and lenses for the optical fibers were adopted from Tovar *et al*. [40]. Additionally, different mold fabrication methods were tested to create a chip with minimal surface roughness and therefore minimal impact on the laser light propagation direction. The lowest surface roughness was achieved on chips from SU8 molds fabricated with soda lime masks (Section S2.2). Furthermore, the impact of the height of the microfluidic chip was analyzed and adapted to reduce light scattering (Section S2.3). Additionally, to prevent fluorescent light from the fluorescence codes and their exciting lasers to impact the ARS images, a filter was placed on top of the imaging detector matrix (Section S2.4 and S2.5).

**Fig. 2:**
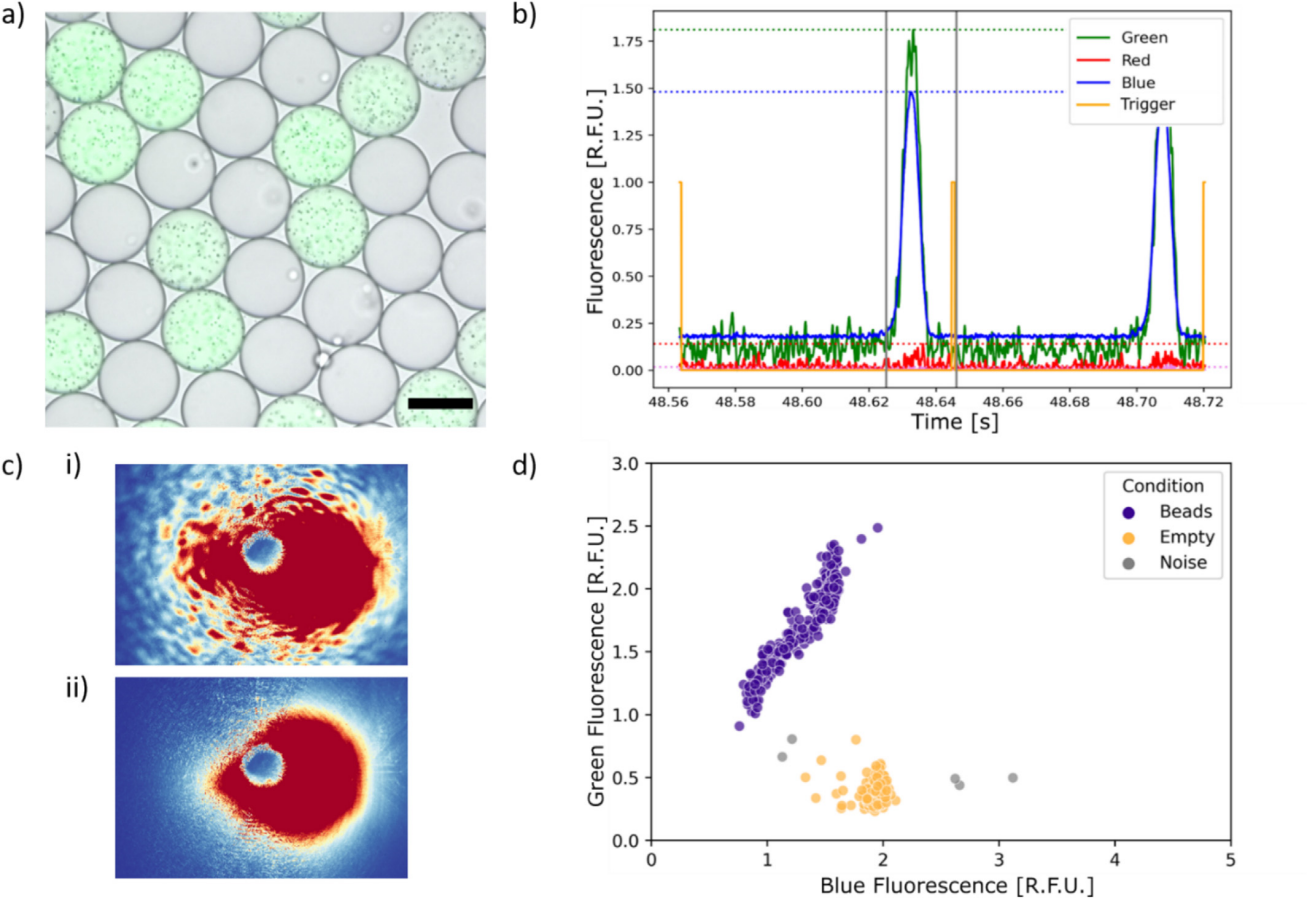
Multiplexed proof-of-principle experiment. a) Two populations of droplets (“empty” droplets with blue fluorescent dye and droplets with beads + blue + green fluorescent dyes) were analyzed. The scalebar represents 50  µm. b) The fluorescent signals within a defined detection window (gray lines) around the trigger signals were analyzed regarding their height (dotted lines) and widths. c) ARS images of a droplet i) with beads and ii) without beads. d) The fluorescence peak signals were plotted and clustered with DBSCAN. All ARS images displayed the droplet condition their corresponding fluorescence signals were clustered in. (For interpretation of the references to color in this figure legend, the reader is referred to the web version of this article.)

In a proof-of-principle multiplex experiment, two distinct droplet populations were created: one with blue fluorescent dye and another with a combination of blue and green fluorescent dyes along with 1  µm sized beads (BLINK DX) (Fig. 2a). These two populations were then combined and examined using our detection platform. The fluorescence signals and ARS images of hundreds of droplets from two technical replicates were recorded at around 20  Hz and analyzed (n = 500 and n = 731). Specifically, we examined the fluorescence peaks regarding their heights and widths within a defined time window around each trigger signal (Fig. 2b) and matched them to the corresponding ARS image based on the recording order.

The ARS images were manually analyzed to determine the presence of beads, which produce characteristic speckle patterns easily distinguishable from those without beads (Fig. 2c). The fluorescence peak values were clustered into two clusters using the machine learning approach Density-Based Spatial Clustering of Applications with Noise (DBSCAN). DBSCAN is a clustering algorithm that groups together points that are closely packed and marks points as noise that lie in sparse regions. Noise points do not belong to any identified clusters and are ignored for subsequent analysis. In addition to correctly identifying two distinct clusters, all signals with high green fluorescence peak values were consistently matched to ARS images of droplets with beads. Conversely, low green fluorescence signals were matched exclusively to images of droplets lacking beads (Fig. 2d and Section S3.1).

This experiment demonstrated that our detection platform could successfully match fluorescence signals with ARS images with an accuracy of 100 percent, thereby confirming its capability for multiplexed experimentation.

### Evaluation of the accuracy of our platform for predicting cell concentrations with CNNs

**Fig. 3:**
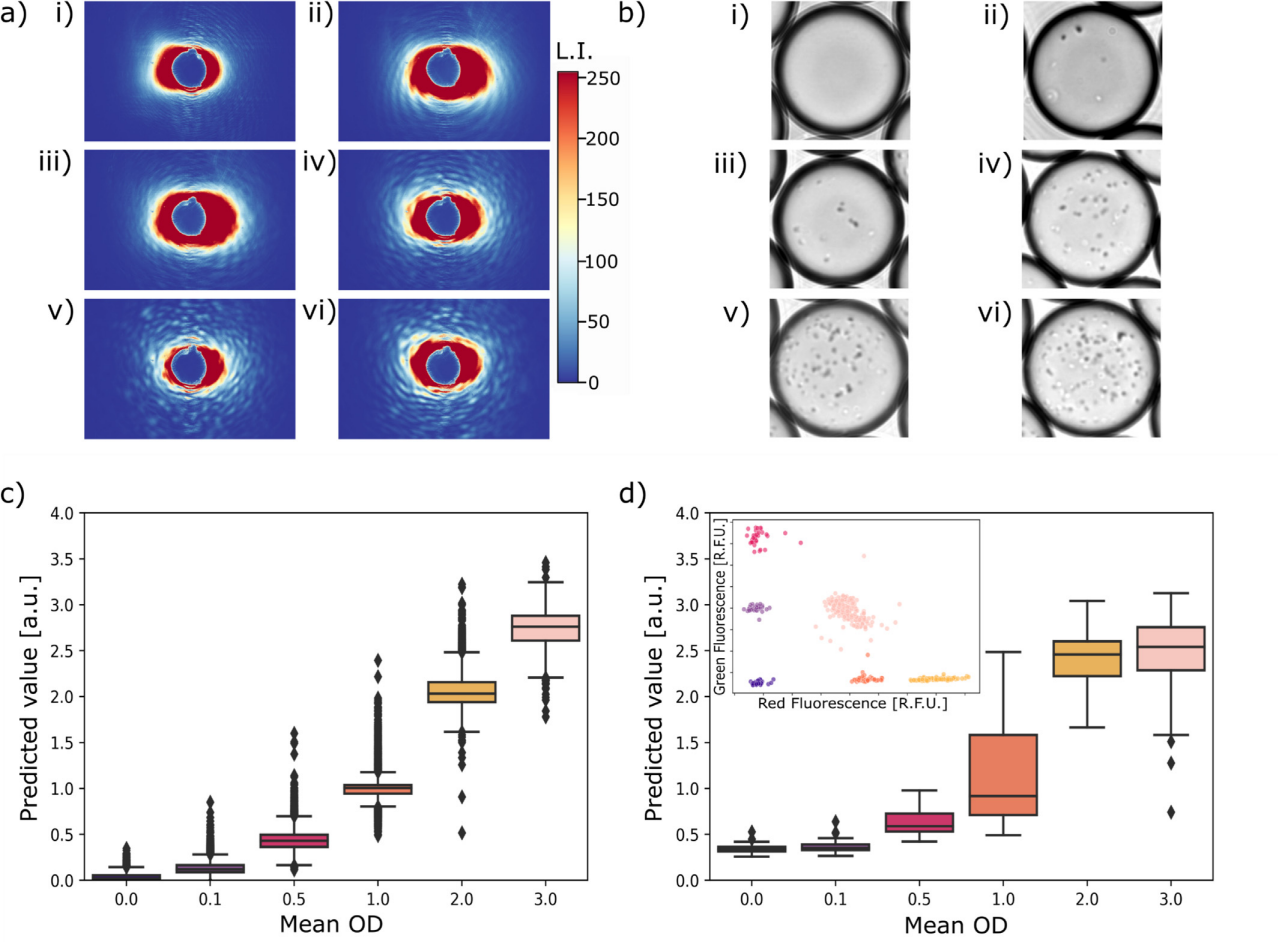
Predicting growth results from single and multiplexed experiments. a) ARS and b) bright field images of droplets containing on average i) 0, ii) 10, iii) 60, iv) 120, v) 240 and vi) 360 cells corresponding to OD 0, 0.1, 0.5, 1, 2, and 3, respectively. c) Prediction results of ARS images from separate measurements for each mean OD (droplets: n = 49,491). d) Prediction results of multiplexed experiment (droplets: n = 1,013). The mean ODs were separated based on the detected fluorescence clusters (inset). L.I. = Light Intensity.

In order to determine cell concentrations from ARS images, a CNN based regression model needed to be trained for prediction purposes. To achieve this, droplets from six different sample ODs (0, 0.1, 0.5, 1, 2, 3) were generated. This resulted in an average of 0, 10, 60, 120, 240 and 360 *S. aureus* cells per droplet [38]. The droplet populations were imaged separately with the ARS sensor to know the average cell concentration displayed in each image (Fig. 3a). Additionally, brightfield images of the droplet populations were taken to ensure correct droplet generation and cell encapsulation (Fig. 3b). The regression model was trained on the ARS images (Material and Methods and Section S3.2) [38]. The predicted values increase as the mean OD increases, and therefore a relative cell concentration can be estimated (Fig. 3c). The spread of the predicted values is caused by the random number of cells encapsulated in each droplet for a given cell concentration.

As a next step, a multiplexed experiment of droplets with defined cell concentrations was conducted to validate our experimental and analysis pipelines. To achieve this, specific concentrations of green and red fluorescent dyes were added to each sample, resulting in a unique fluorescence code for each droplet population derived from the different ODs. Following droplet generation, the populations were mixed and analyzed using our fluorescence detection and ARS imaging platform. In order to analyze a large number of droplets per condition, an imaging frequency of approximately 40–50 Hz (2400–3000 droplets scanned within one minute) was set, as a trade-off between high-throughput and minimizing the error during ARS image acquisition. If the time interval between two image acquisitions was too short, the data transmission from the camera to the PC and/or the saving of the image data could not be conducted due to large image sizes, limited data transfer and/or computational limitations. As a result, images were dropped. To deal with these dropped images, a python-based analysis pipeline was developed to match the fluorescence signals to the images since they were captured by two different systems (Section S3.3).

To distinguish the six fluorescence codes, the fluorescence peak signals were clustered. The relative fluorescence intensities of these clusters correlate with the concentrations of the fluorescent dyes within the droplets. The ARS images matching to the six identified clusters were analyzed using the CNN-based regression model, which demonstrated a positive correlation between the predicted values and the increasing mean OD (Fig. 3d). Even though the differences between the droplet populations are less pronounced than in the separate analysis, our integrated platform can distinguish between different cell concentrations within droplets for multiplexed and high-throughput experiments. Our CNN classifiers and regressors were trained exclusively on *S. aureus* droplets across defined OD ranges. Although these models can generalize to recognize growth-related morphological changes in other species, their quantitative accuracy on non-*S. aureus* samples has not been validated. Application to a different bacterial strain or species will therefore require fine-tuning on a small, species-specific dataset or full retraining with multi-species data before reliable use in new contexts.

### Multiplexed AST with various *S. aureus* strains

Four different *S. aureus* strains, with known antibiotic resistance profiles were tested against five antibiotics (PEN, GEN, CIP, ERY, and TET) at the clinical breakpoint concentrations defined by the European Committee on Antimicrobial Susceptibility Testing (EUCAST, version 2023). Clinical breakpoints are antibiotic and organism specific antibiotic concentrations used to define whether the infection can be treated with that antibiotic. If the organism’s growth is inhibited when exposed to a clinical breakpoint concentration, it is classified as susceptible (S), however, if it is able to grow, then it is classified as resistant (R). An organism can also be deemed as “susceptible, increased exposure” (I), when there is a high probability of therapeutic success if the exposure to the antibiotic is increased by adapting the dosing regimen or by adjusting the antibiotic concentration at the site of the infection. If an organism can be deemed as “I” to an antibiotic, then two breakpoint concentrations exist. Out of the five tested antibiotics, only CIP has two breakpoint concentrations, however, only the lower concentration was used to determine susceptibility.

To achieve multiplexed experimental conditions for rapid AST, single cells were encapsulated with an antibiotic and fluorescent dyes to create a specific color code for each antibiotic. The droplets were then incubated at 37 °C for five hours and analyzed hourly. To prevent interdroplet transfer of the antibiotics, the different droplet populations were mixed just before ARS image acquisition (Section S4) [46,47].

For each strain and timepoint, around 20,000 to 40,000 droplets were characterized. The predicted ODs of each antibiotic conditions at the different timepoints were plotted against droplets containing only media (“empty”) and droplets containing cells but no antibiotic (“w/o ab”) for each tested *S. aureus* strain. When targeting a single cell per droplet, the majority of droplets remain empty. To prevent droplets with no cells from obscuring the visual interpretation of the data, the expected empty fraction of droplets for each antibiotic condition as well as for the “w/o ab” population were removed for plotting (Fig. 4 and Section S5).

Overall, the predicted values for each condition resemble consistent growth curves, indicating that the data synchronization and analysis was successful. An increase in growth in the “w/o ab” population can be visually detected for three strains after a single hour and for one strain after three hours of incubation.

In order to determine, when the predicted ODs of the antibiotic conditions are significantly different to the control population without antibiotic, a statistical model was developed. For this, a threshold was determined that divides the droplets into droplets with no cell growth and droplets with cell growth. Afterwards, the OD distribution of droplets with growth of each antibiotic condition was compared to the OD distribution of droplets with growth of the control population “w/o ab” at each timepoint. If the distributions are completely identical, a value of 0.5 is received. The more the value deviates from 0.5, the greater the OD distributions differ. Cells were considered as susceptible to an antibiotic if a value of >= 0.8 was achieved. A value < 0.8 showed significant growth and was interpreted as resistance of the strain to this antibiotic. Values <= 0.2 indicated an even higher growth with the antibiotic than without. The results of this statistical approach are shown in Fig. 5 for all four tested strains together with the AST results from VITEK 2 and the gold standard disc diffusion method to validate our developed platform.

Our platform has a 95 % categorical agreement after three hours of incubation with the results derived after 20 h of incubation with the disc diffusion technique (Fig. 5 and Section S6). Furthermore, our platform showed the same level of categorical agreement with the disc diffusion results as VITEK 2 which needed 8–14 h for analysis. Only one misclassification occurred, as strain 4 was falsely classified as susceptible against CIP. Since only the lower breakpoint concentration for CIP was used, the classification was limited to S and not-S for CIP. Thus, if no significant change in growth behavior with CIP was observed compared to the control, the strain could be either I or R. For further classification into I and R, strains can be additionally tested against the higher breakpoint concentration of CIP using this platform.

We should note, that maybe a reliable identification of antibiotic susceptibilities would be expected to be possible at earlier time points for bacteriocidal in contrast to bacteriostatic antibiotics. However, in this initial study, TET as a bacteriostatic antibiotic, also allowed for a fast calling of susceptibility after only 3 h. In future work, this should be evaluated carefully for other bactiostatic antibiotics.

The implications for clinical practice are substantial, as this platform could enable personalized treatment regimens to be initiated on the same day, reducing reliance on broad-spectrum antibiotics and therefore help in the fight against the increase of antibiotic resistances. Antibiotic breakpoint analysis is essential in antibiotic stewardship, providing physicians critical information for treatment decisions.

In this study, our platform was utilized exclusively for breakpoint analysis. However, we have previously demonstrated the capability of 2D ARS imaging to assess susceptibility across varying concentrations of the same antibiotic [38]. The platform also has the potential for multiplexed MIC determination of different antibiotics, as it can generate and detect a wide array of fluorescence codes [48]. Currently, the multiplexing capacity is, however, limited by interdroplet transfer of certain antibiotics (see SI Section 4.3), which requires the separate generation and incubation of droplet populations. Future research should focus on identifying an oil-surfactant combination that prevents this transfer, thereby enhancing the platform's scalability.

Other developed phenotypic methods for AST also offer rapid results, sometimes within two hours, such as Resistell, which measures the nanomotion of cells [49], dropFAST, which utilizes the redox indicator dye resazurin for growth detection in microfluidic droplets [50], and iFAST, which measures the electrical properties of cells [51]. But of these strategies only Resistell has been commercialized to this point and it is not known to us if multiplexing of AST testing is established for any of these methods. Pheno-molecular and molecular methods can reduce the time even further to within 15 to 30 min [52,53]. However, our platform combines multiple advantages: (i) a label-free detection of cells ensures simple and fast sample preparation, (ii) susceptibility identification based on actual growth within a short timeframe of three hours, (iii) high-throughput characterization of tens of thousands of samples to antibiotics at the single-cell level in one single experiment, and (iv) multiplexed experimentation enables the testing of multiple antibiotic conditions and various bacterial samples at once.

## Conclusion

This work demonstrated the successful development of a rapid, multiplexed and high-throughput AST platform that combines label-free 2D ARS imaging for microbial growth detection with fluorescence detection for droplet condition identification. *S. aureus* strains with various antibiotic resistance profiles were used to showcase the platform’s capabilities. This platform enables accurate decoding of fluorescence codes and determines relative cell concentrations from ARS images based on CNN regression models. Crucially, the system maintains the integrity of image and fluorescence signal matching, even when operating at higher flow rates where image dropping occurs. Notably, the platform delivers a resistance/susceptibility profile within three hours of incubation, a significant time reduction compared to the traditional 8–20 h incubation period required by the gold standard phenotypic AST methods used in clinics. This rapid turnaround was validated against the broth microdilution method using VITEK 2 and the disc diffusion method. Earlier detection, such as after one or two hours of incubation, remains limited by the lag phase of some microbial strains.

We have shown in our previous work that the entire scattered light detection and data analysis platform works just as well for early detection of *E. coli*. Next development steps of this platform should therefore expand the AST testing to other pathogens, which might also include anaerobic microorganisms. Thus, for a further development towards clinical applications, the applicability of the platform to a wide range of pathogens, an evaluation of applicability for slow versus fast growing microorganisms, and requirements for sample preparation have to be targeted in follow-up research.

Beyond AST, this versatile platform holds promise for a variety of applications, including population heterogeneity studies, growth condition optimization, and the detection of rare and slow-growing microorganisms from environmental samples.

## CRediT authorship contribution statement

**Martina Graf:** Conceptualization, Formal analysis, Investigation, Methodology, Visualization, Writing – original draft, Writing – review & editing. **Arjun Sarkar:** Formal analysis, Methodology, Writing – review & editing. **Carl-Magnus Svensson:** Formal analysis, Methodology, Writing – review & editing. **Anne-Sophie Munser:** Methodology, Writing – review & editing. **Sven Schröder:** Supervision, Writing – review & editing. **Elke Mülle:** Methodology, Writing – review & editing. **Sundar Hengoju:** Supervision, Writing – review & editing. **Marc Thilo Figge:** Funding acquisition, Resources, Supervision, Writing – review & editing. **Miriam A. Rosenbaum:** Conceptualization, Funding acquisition, Resources, Supervision, Writing – review & editing.

## Declaration of competing interest

The authors declare the following financial interests/personal relationships which may be considered as potential competing interests: M.G., A.S., C.M.S., A.S.M., S.S., M.T.F., M.A.R have applied for a patent for the 2D ARS imaging and analysis platform (“Optische Messvorrichtung, Messverfahren und Auswerteverfahren“, Patent pending, 10 2024 103 308.8, 2024).

The remaining authors declare no conflict of interest.
