# Supplementary material for: Multiplexed, rapid phenotypic antibiotic susceptibility testing based on angle-resolved light scattering imaging of microfluidic droplets
Source: J Adv Res. 2025 Sep 25;84:405–14. doi: 10.1016/j.jare.2025.09.047 (PMC13227228; doi:10.1016/j.jare.2025.09.047)
Supplement: Supplementary Data 1 [file mmc1.docx]

Supplementary Information

**Multiplexed, rapid phenotypic antibiotic susceptibility testing based on angle-resolved light scattering imaging of microfluidic droplets**

*Martina Graf, Arjun Sarkar, Carl-Magnus Svensson, Anne-Sophie Munser, Sven Schröder, Elke Müller, Sundar Hengoju, Marc Thilo Figge, Miriam A. Rosenbaum*

**Contents**

[1. Setup of platform 1](#_Toc181103216)

[2. Optimization of platform 2](#_Toc181103217)

[3. Multiplexing 6](#_Toc181103218)

[4. Interdroplet transfer of antibiotics 12](#_Toc181103219)

[5. AST results of all experiments 18](#_Toc181103220)

[6. Disc diffusion results 27](#_Toc181103221)

[References 27](#_Toc181103222)

# **Setup of platform**


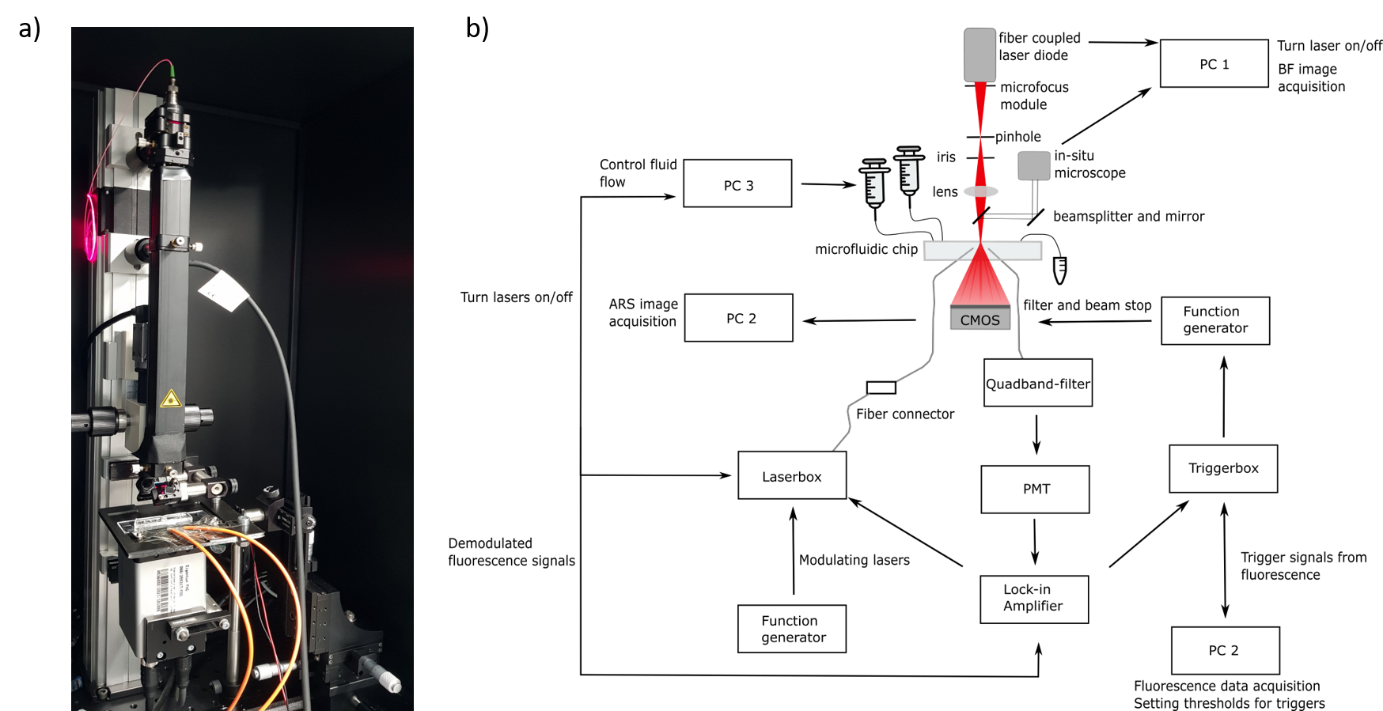


**Figure S1** a) Picture of the ARS sensor. b) Setup of the fluorescence detection and ARS imaging platform

# **Optimization of platform**

**2.1 Chip design**

The microfluidic chip designed and used for all experiments is displayed in Figure S2. For optimal fluorescence detection and ARS imaging there were several things that had to be considered for the chip design:

1. The droplets needed to be able to flow through a microfluidic channel without being squeezed by the vertical walls of the channel. The resulting droplet deformation, will cause the droplets to be much bigger than the ARS laser spot size and therefore a great part of the droplets and cells will not be illuminated. But more importantly, if the vertical channel walls are also illuminated, they will also cause the laser light to scatter, adding an additional level of complexity onto the resulting ARS scatter pattern and decreasing the sensitivity towards detecting cellular growth. A channel width of 75 µm was chosen at the fluorescence detection and ARS image acquisition site, since droplets had a diameter of around 55-60 µm.
2. The spacing of the droplets is crucial for signal recording and imaging of single droplets. If droplets are too close to each other, the second droplet can impact the recorded fluorescence signals and the scattering pattern of the first droplet. If they are too far apart, the acquisition speed will be low. Therefore, two oil inlets were designed for finetuning the droplet speed and spacing.
3. Fluorescence detection: The fiber guiding structures and lenses were taken from Tovar *et al.*^[1]^ For optimal detection, the droplets need to be illuminated by a fiber perpendicular to the channel and the emitted fluorescence is detected in an angle to the microfluidic channel.
4. ARS imaging: A long straight channel after the fluorescence detection site enables the ARS image acquisition at different droplet speeds and with various waiting times in between fluorescence detection and triggering of camera. The shortening or prolonging of waiting times was very helpful in finding a spot for ARS imaging with very minimal background noise.


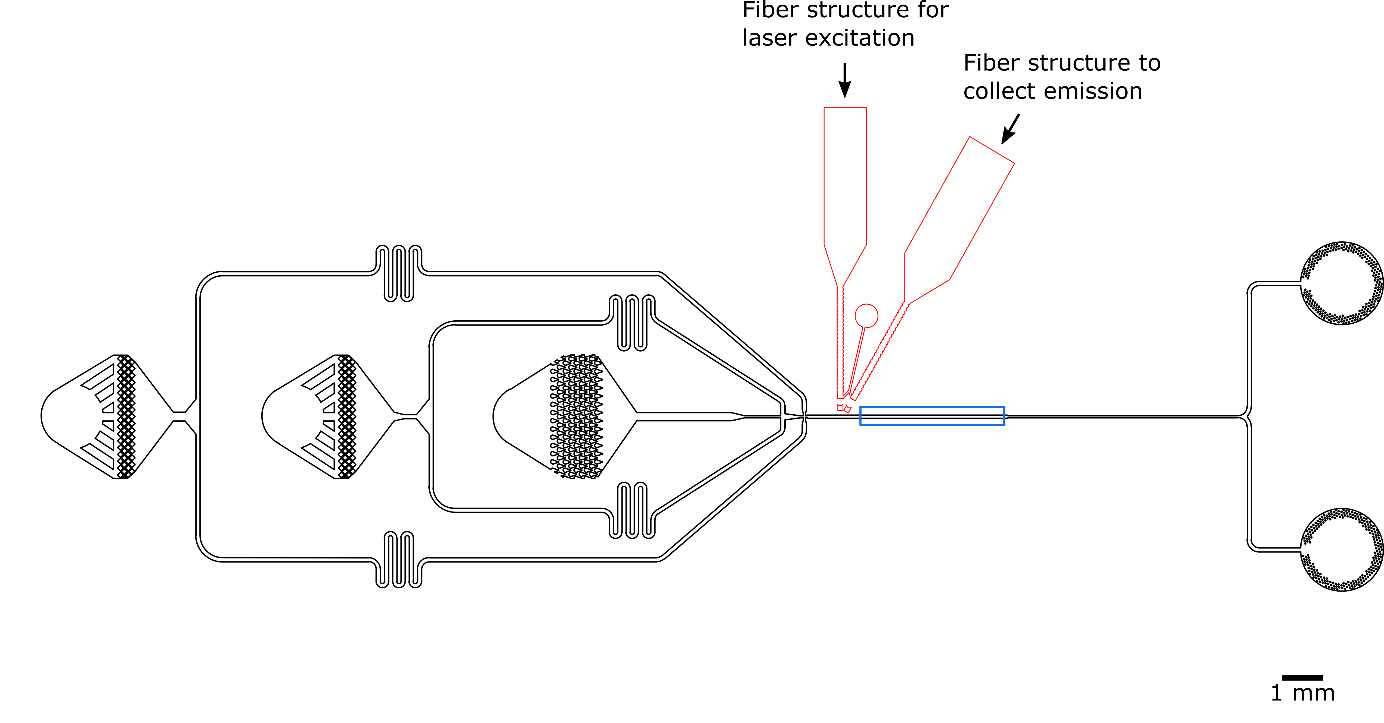


**Figure S2** Microfluidic chip layout. In black are the fluid channels and in red the channels used for fiber integration. The blue box marks the area in which ARS imaging is conducted. The exact spot for ARS imaging varies.

**2.2 Droplet uniformity**

For quantifying droplet size variability, microscopic images of static droplets were obtained and analyzed using ImageJ. Minimal variation in droplet size was observed with a coefficient of variation (CV) of less than 6.7% for six different droplet populations containing different concentrations of cells (from Figure 3). Table S1 summarizes the mean area of droplets (in pixels).

**Table S1** Exemplary size variability analysis of droplets with different cellular biomass from Figure 3.

| **Sample (OD)** | **Mean** | **Std** | **CV** | **Count** |
| --- | --- | --- | --- | --- |
| 0 | 1752.229 | 75.04498 | 4.28283 | 205 |
| 0.1 | 1862.574 | 124.7209 | 6.696156 | 175 |
| 0.5 | 1654.728 | 57.15647 | 3.454132 | 245 |
| 1 | 1979.303 | 126.6714 | 6.399801 | 237 |
| 2 | 2033.286 | 67.79956 | 3.334482 | 181 |
| 3 | 2030.338 | 49.79298 | 2.452448 | 269 |

The same droplet populations from Figure 3 were also used to access content uniformity, as they all contained equal amount of blue fluorescent dye. A very low CV of less than 2.3% was observed confirming uniform content for different droplet populations. The detailed values for blue fluorescence intensity (in volt) are shown in Table S2. Thereby, the fluorescence signal fluctuations are independent from CNN models. The CNN model was used for analyzing ARS images. After completion of CNN analysis, data were merged using python script.

**Table S2** Accessing droplet content uniformity through the analysis of the blue fluorescence signal

| **Sample (OD)** | **Mean** | **Std** | **CV** | **Count** |
| --- | --- | --- | --- | --- |
| 0 | 4.800937 | 0.10694 | 2.227483 | 47 |
| 0.1 | 4.883399 | 0.084993 | 1.740444 | 44 |
| 0.5 | 4.843143 | 0.045275 | 0.934825 | 37 |
| 1 | 4.950358 | 0.061675 | 1.245878 | 58 |
| 2 | 5.101474 | 0.066683 | 1.307129 | 94 |
| 3 | 5.098353 | 0.106594 | 2.090759 | 231 |

**2.3 Surface roughness measurements**

Different fabrication methods for the chip molds were tested to see which resulted in the lowest surface roughness. A high surface roughness impacts the scattering pattern significantly and therefore reduces the capability of detecting few bacterial cells. White light interferometry with a 50 x magnification was used to determine the surface roughness. The root mean square (rms) of three measurements of each mold was calculated. Unsurprisingly, the femtoprinted mold had the highest rms value and resulted in a high background noise on the ARS image. SU8-molds had much lower rms values, with a soda lime mask manufactured mold having even a lower surface roughness (rms = 2.8 nm) than the mold fabricated with a film mask (rms = 9.3 nm) (Figure S3a). The impact on the ARS image was much lower with chips made from SU8-molds (Figure S3b), therefore chips made with soda lime masks were used for all experiments.


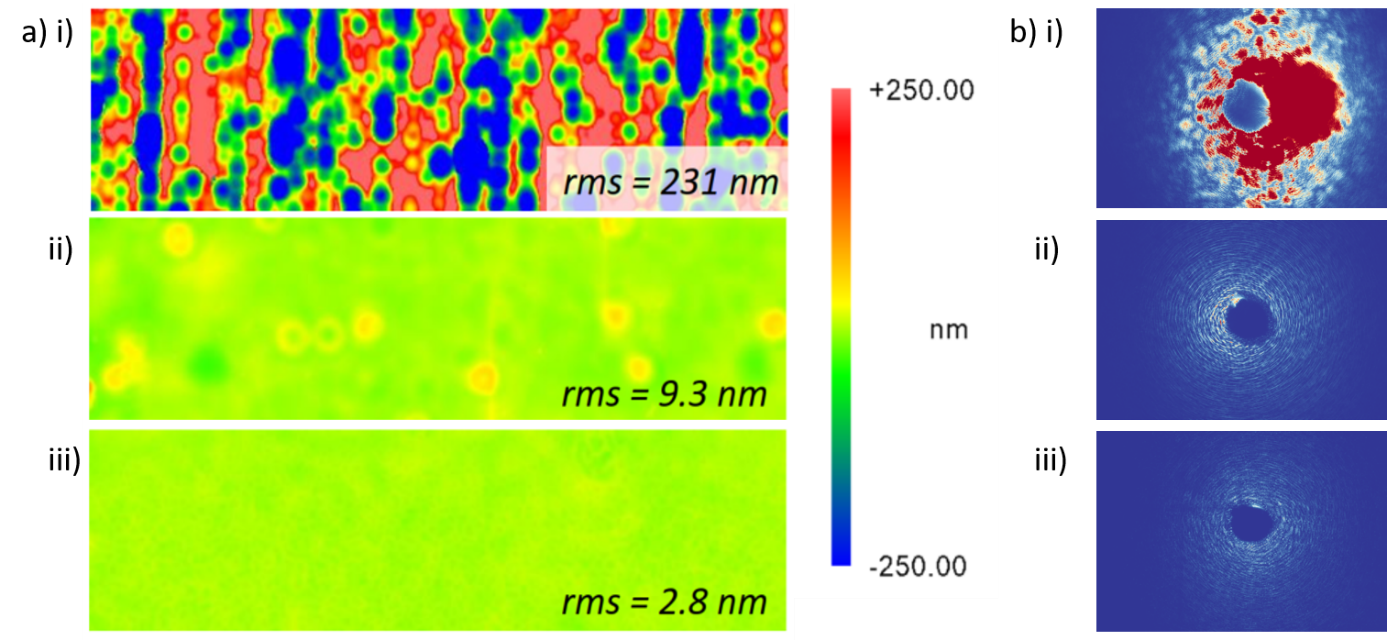


**Figure S3** a) Surface roughness measurements of chips that were manufactured from i) 3D glass mold ii) SU8 mold from film mask iii) SU8 mold from soda lime mask. The root mean square (rms) of three measurements per chip was taken. b) ARS images of chips manufactured from i) 3D glass mold ii) SU8 mold from film mask iii) SU8 mold from soda lime mask.

- 1. **Impact of PDMS height on the chip**

Chips with different PDMS heights were tested to analyze the impact of the PDMS height on the ARS image. Thicker chips caused more background noise than a very thin layer of PDMS. The very thin layer (< 0.5 mm) caused barely any background noise (Figure S4), however, the manufacturing process is more time consuming and therefore it was decided to use chips with a height of around 3 mm as a trade-off between background noise and easy manufacturing.


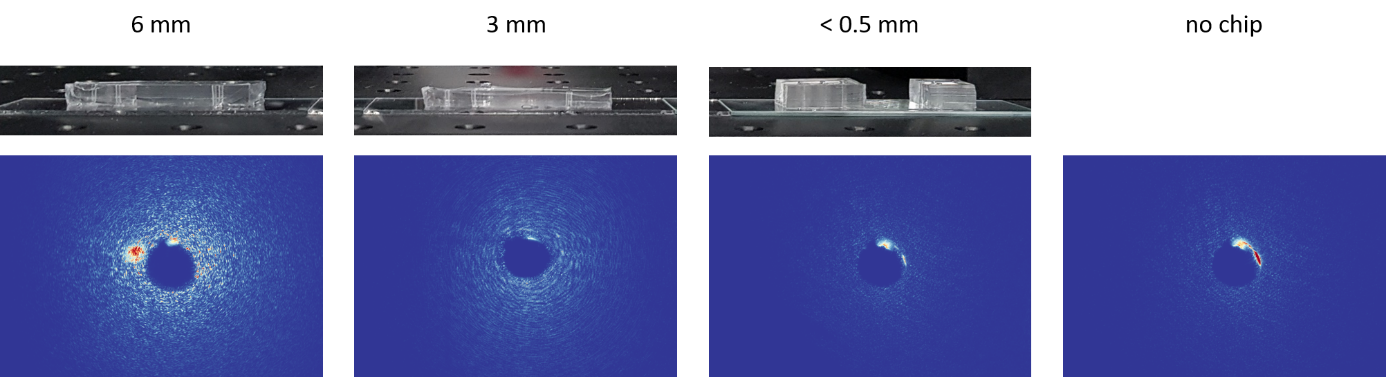


**Figure S4** Impact of PDMS height of microfluidic chip onto the ARS image. The thicker the chip, the more the laser light was scattered, resulting in a higher background noise on the ARS image.

- 1. **Laser filter test**

The lasers used for detecting the barcodes of the droplets will have an impact on the ARS images. Therefore, a filter was needed in front of the ARS sensor to block out the wavelengths caused by the barcoding lasers. Three different filters were tested: FELH650, FGL645 and FGL630 with cut-on wavelengths of 650, 645 and 630 nm, respectively. ARS images were always taken with just the ARS laser on and with all lasers on. The mean grey values of the images were determined as a measurement of impact of the barcoding lasers on the images. When imaging without a filter, a strong difference in the mean grey values between the barcoding lasers being on or off is visible. This shows the necessity of a filter on top of the detector matrix to increase sensitivity. With a filter, regardless which of the three versions tested, no significant difference was seen when the barcoding lasers were on or off. Therefore, the emission wavelengths of the dyes were successfully filtered out. However, the mean grey values of the ARS images differed for the different filters in comparison to not having a filter at all and only the ARS laser on (Figure S5). Based on that, the filter FGL630 was chosen.


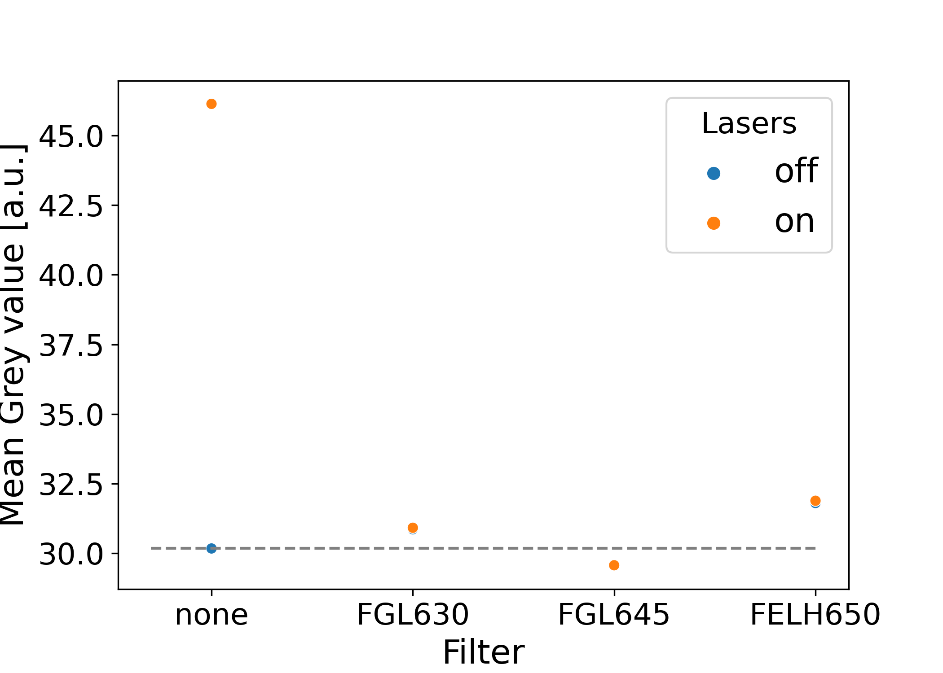


**Figure S5** Laser filter test. Three filters were compared to operation without filter to find the best for the application. The ARS laser was turned on for all measurements. The lasers for detecting the fluorescence signals were either on or off. The mean grey values between the fluorescence lasers being on or off are almost identical when filters were used (blue scatter point is mostly covered by the orange scatter). Grey dotted line highlights the mean grey value of an ARS image without the fluorescence detection setup.

- 1. **Visualization of the impact of the different objects in the laser path on the ARS images**


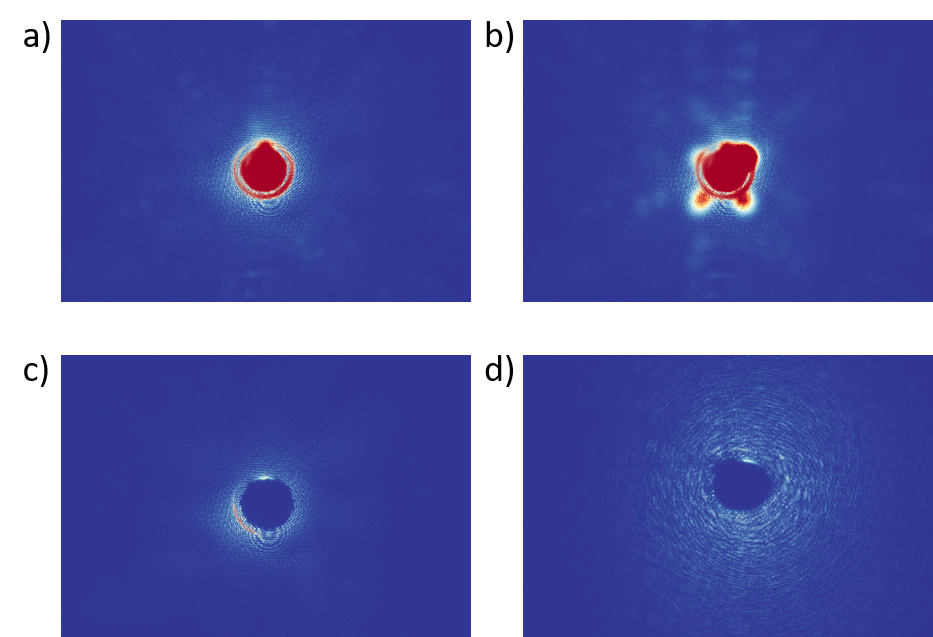


**Figure S6** Example ARS images with different objects in the laser path. The lasers used for coding are turned off. a) nothing in the laser path, b) filter FGL630 on top of the detection matrix, c) filter and beam stop on top of the detection matrix, d) filter, beam stop and microfluidic chip in the laser beam. A beam stop is important to reduce reflection and other interference patterns caused by directly transmitted light.

# **Multiplexing**

**3.1 proof-of-principle experiment**

The accuracy of matching the fluorescence and ARS images was validated by clustering the fluorescence data and manually analyzing the corresponding ARS images (Figure 2 and S7). The fluorescence peaks were identified by analyzing the fluorescence signals between 0.02 s before and 0.001 s after each trigger signal. For the analysis with DBSCAN, the parameters eps = 0.3 and min_samples = 10 were used.


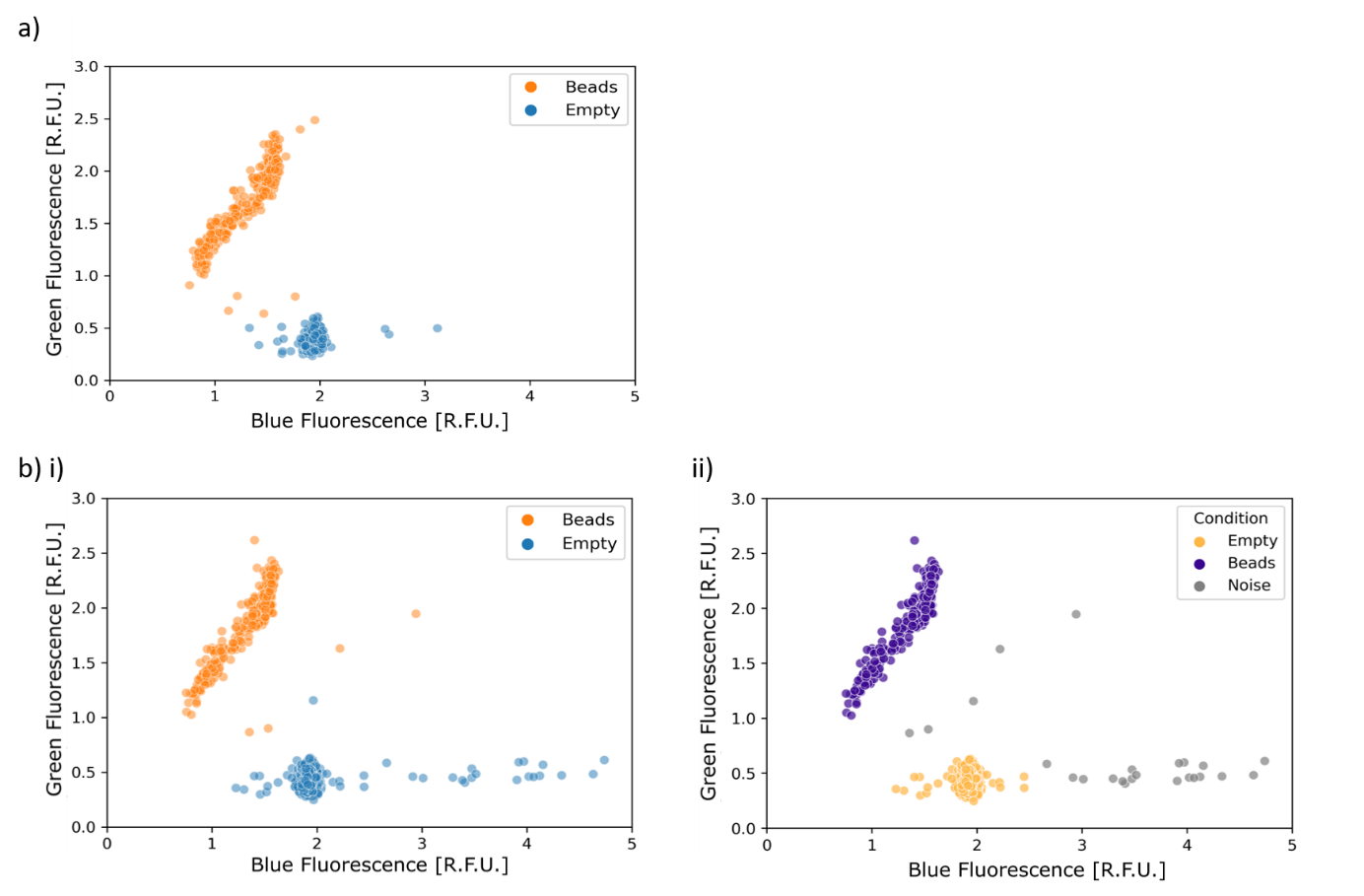


**Figure S7** Multiplexed experiment with two droplet populations containing either green and blue fluorescence dyes in addition to around ten 1 µm sized beads (beads) or just blue fluorescence dye (empty). a) Fluorescence peak values and their corresponding droplet population (beads or empty) based on ARS images are displayed for replicate 1 (n= 500) that is also described in Figure 2 in the main text. b) i) same as a) but for replicate 2 (n= 731). b) ii) The machine learning algorithm DBSCAN was used to automatically classify the fluorescence signals from i). The classification had an accuracy of 100 %, meaning that all ARS images with beads were classified as containing beads based on the fluorescence data and all empty droplets were classified as empty. Black scatter points are noise and are not classified as either empty or with beads.

**3.2 ARS analysis with CNNs**

ARS images were available for both 400 µs and 800 µs exposure times to train deep learning algorithms for predicting bacterial growth. However, some images in the dataset were falsely triggered, either missing the droplets entirely or capturing them as they exited the field of view. To maintain the integrity of the deep learning training process, it was crucial to filter out these inaccurately captured images. To accomplish this, 3,222 correctly triggered images and 3,222 falsely triggered images were manually separated. To augment the dataset, each image was horizontally flipped, resulting in a total dataset size of 12,888 images (6,444 images per set).

A CNN model based on EfficientNetB4^[2]^ was employed to classify these images, utilizing transfer learning with pretrained ImageNet weights. Of the 12,888 images, 20% were set aside for testing. From the remaining 80%, 20% were used for model validation, and the rest were used for training. The trained model, which was subsequently saved, achieved an overall accuracy of 99.0%, demonstrating its effectiveness in accurately classifying and filtering the ARS images.


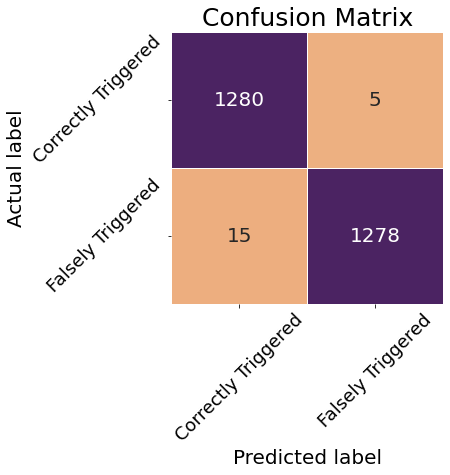


**Figure S8** Confusion matrix of correctly triggered and falsely triggered images on the test set

After filtering out all falsely triggered images, a total of 106,490 images remained, corresponding to the following optical density (OD) values: 0, 0.1, 0.5, 1, 2, and 3. The dataset contained an uneven number of images for each OD value. To balance the training dataset, 9,000 images of each OD value were selected, resulting in a total of 54,000 training images. Additionally, 3,000 images were set aside for validation, and the remaining 49,490 images were used to test the model.

Several data augmentation techniques were applied to the training dataset, including horizontal and vertical flipping, rotation, and shearing, to enhance the model's robustness. An EfficientNetV2-XL model^[3]^, pretrained on the ImageNet21k dataset^[4]^, was utilized as the feature extractor. This was followed by a fully connected layer with 1,024 nodes and ReLU activation^[5]^, and a final layer with a single node using linear activation for regression. The model was trained using the Adam optimizer^[6]^ with a mean squared error (MSE) loss function. Upon completion, the trained model was saved. The model achieved very good performance metrics, including a mean absolute error (MAE) of 0.094, an MSE of 0.021, a root mean squared error (RMSE) of 0.143, and an R² value of 0.971.

For testing on all multiplexed ARS images, the falsely triggered images were first removed using the previously trained model. Subsequently, each remaining image was processed through the trained CNN regression model for prediction.

**3.3 Analysis pipeline for all other experiments**

The analysis pipeline had to be adapted for matching the fluorescence data and the ARS images to be able to deal with dropped images (Figure S9). The first few analysis steps, reading out the timestamps of signals and images as well as detecting the trigger signals and their corresponding fluorescence peaks and widths remained the same. The relative times starting from the first trigger and the first image were calculated separately. The trigger signal with the smallest distance to the first image was matched to the image, along with the corresponding fluorescence peaks and widths. The relative timestamps were set to zero at the matched trigger-image pair. Then again, the closest trigger signal with its corresponding fluorescence data was matched to the next image and the timestamps were zeroed. This was done until every image was matched. However, images that were acquired within a very short time interval of each other were ignored because they were prone to lead to mismatches (Figure S10).


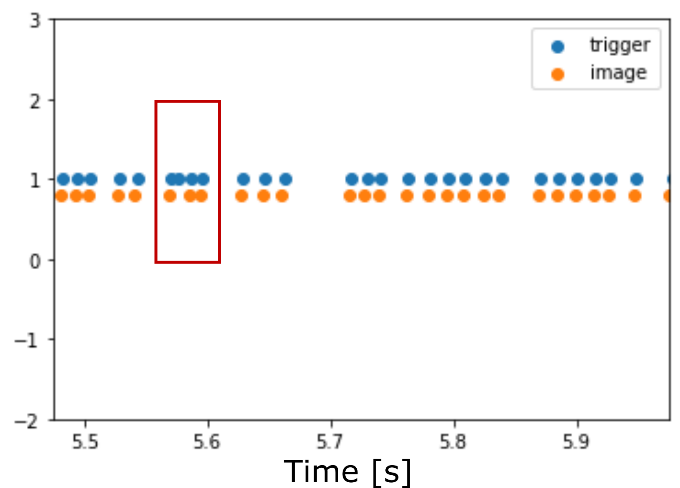


**Figure S9** Triggers and ARS images are plotted over time. The time was set to zero for the first trigger signal and image. Inside the red rectangle, four triggers activated the camera but only three images were saved.

**
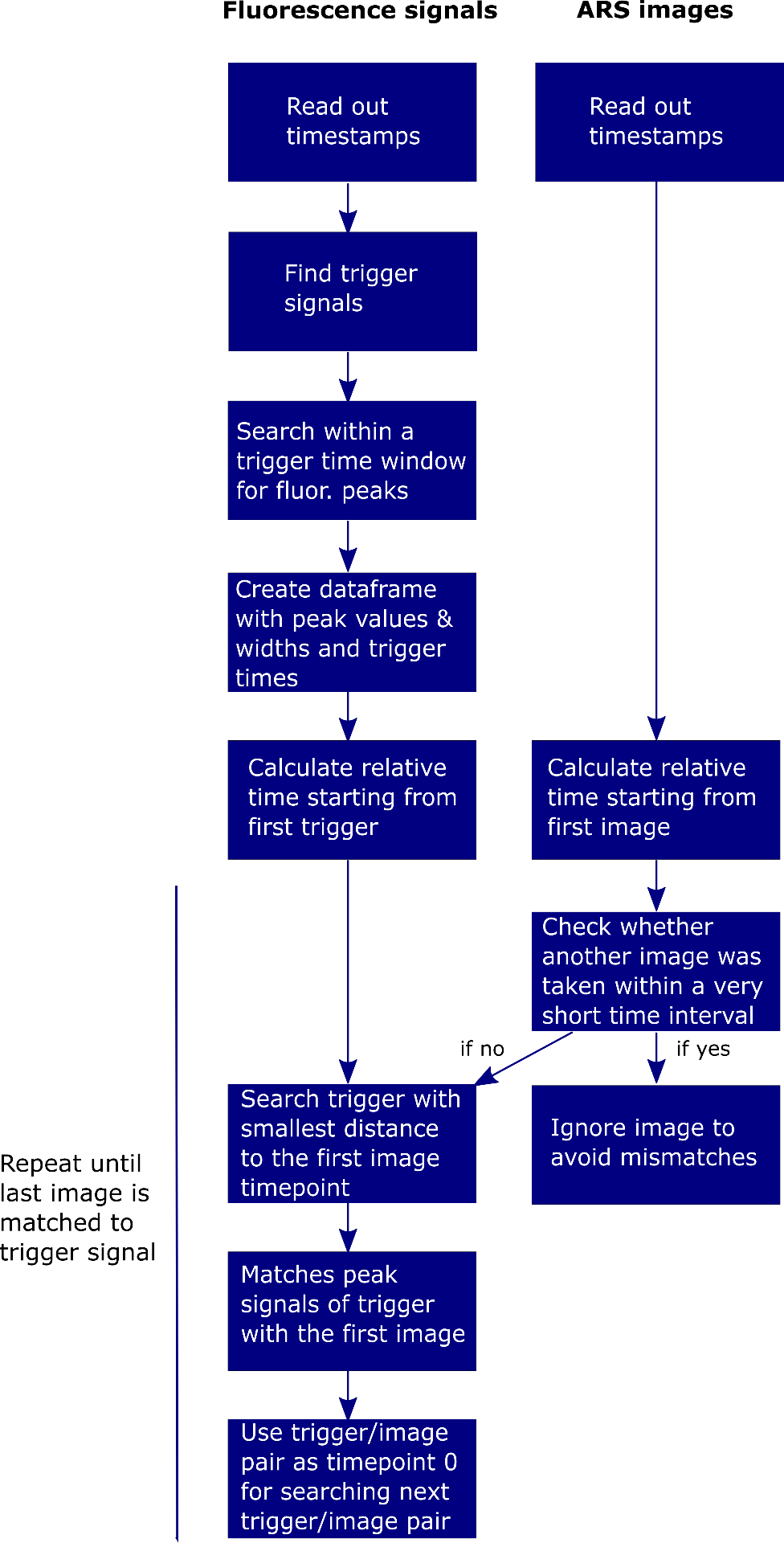
**

**Figure S10** Analysis pipeline for matching the fluorescence data to the ARS image of the same droplet.

# **Interdroplet transfer of antibiotics**

**4.1 Testing four antibiotics for interdroplet transfer**

We observed that growth was inhibited in the control droplets of the multiplexed AST experiment, in which the different antibiotic conditions were incubated together (Figure S11). To check whether the leakage of an antibiotic from one droplet to another caused the inhibition of growth, *S. aureus* droplets without (control) and with antibiotics at their breakpoint concentrations were incubated separately and each antibiotic condition together with control droplets. If the growth inside the control droplets was inhibited when incubated together with an antibiotic, but not when incubated separately under otherwise the same conditions, then it can be assumed that interdroplet transfer of that antibiotic has occurred. Droplets with antibiotics were coded with fluorescent dyes to differentiate from the control droplets. Figure S12 shows, that the growth in the control droplets was less when incubated with TET and ERY but no obvious change in the growth behavior was seen when incubated with GEN and PEN. This means, that a high enough concentration of TET and ERY must have leaked into the control droplets to impact growth. No certain conclusion can be drawn about GEN and PEN. They could have leaked as well but without reaching inhibitory concentrations.


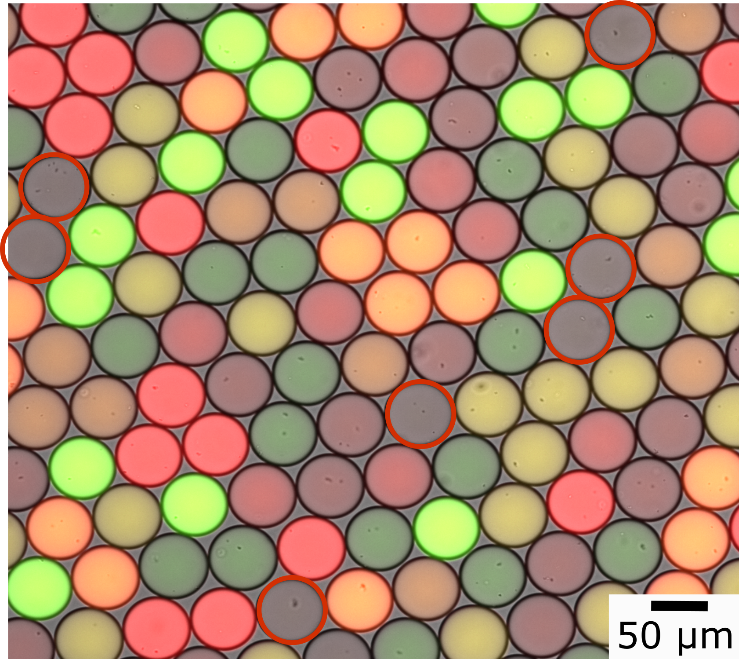


**Figure S11** Droplets with seven different antibiotic conditions marked with fluorescent dyes after four hours of incubation. Growth was inhibited in all droplets including the control condition without any antibiotic (red circled droplets). Image was taken with 20x magnification.


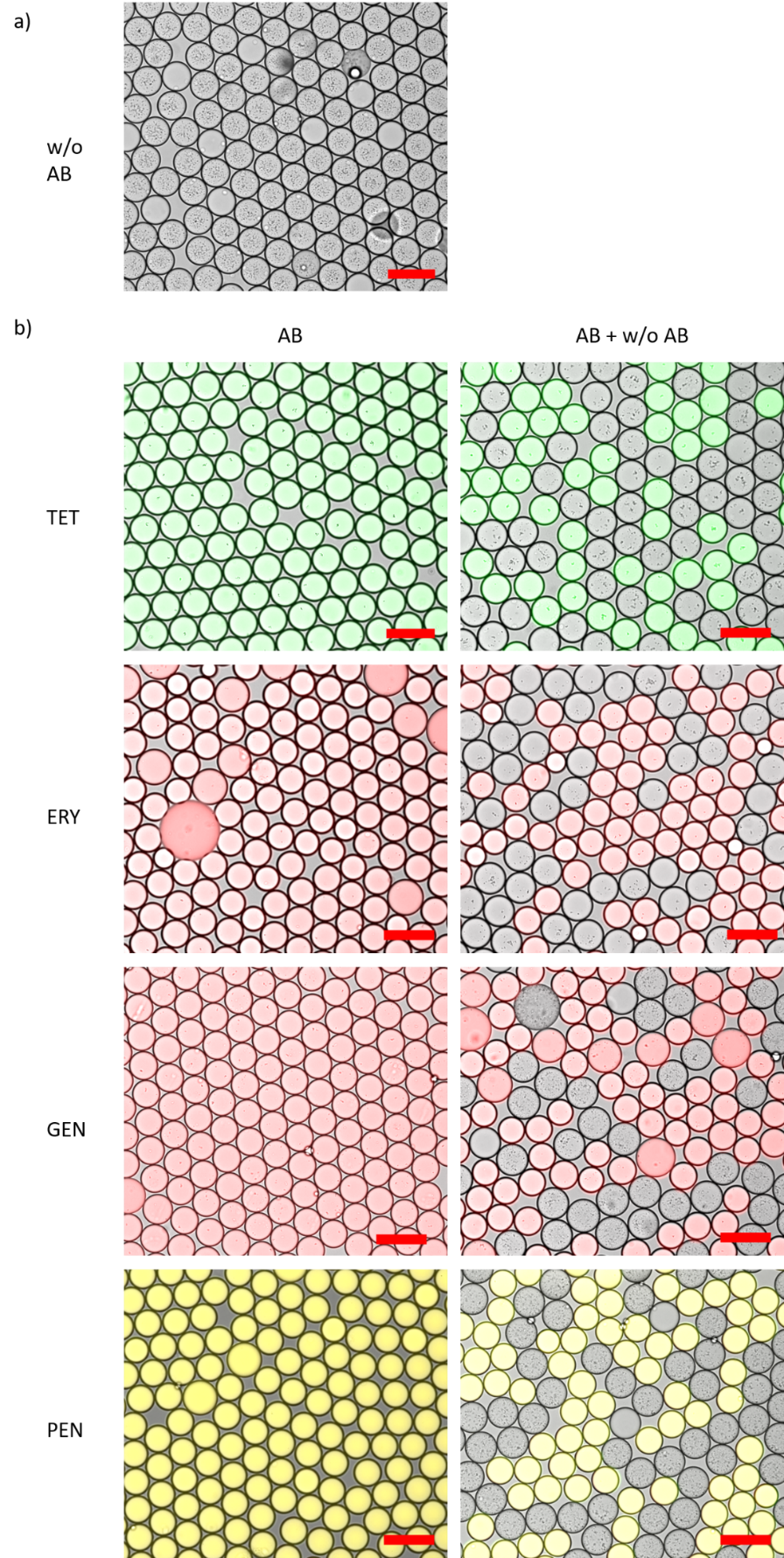


**Figure S12** Interdroplet transfer test of four antibiotics. a) Brightfield image of droplets with S. aureus cells after 5 h of incubation. b) Brightfield and fluorescence images of droplets with S. aureus and four antibiotics (colored droplets) after 5 h of incubation. The same antibiotic concentrations were used as for the AST experiments in the main text. The scalebars represent a length of 100 µm. Images were taken with 20 x magnification.

**4.2 Effect of incubation strategy on interdroplet transfer**

As a next step, we analyzed whether a different incubation strategy can minimize interdroplet transfer. Before that, a static incubation strategy was followed. Meaning, that droplets were placed together with surfactant containing oil inside of microreaction tubes and stored at 37 °C, without any agitation or oil recirculation. As an alternative strategy, a dynamic approach as described by Mahler *et al*. has been tested^[7]^. In this strategy, oil is permanently flushed through the droplets during incubation, ensuring a continuous supply of oxygen to the droplets. But also, the droplets are more separated from each other causing less direct droplet-to-droplet contact with this method. ERY was used for comparing interdroplet transfer with both incubation strategies.

Single *S. aureus* cells were encapsulated without ERY and with 1 µg/mL ERY (coded with red fluorescence dye) (Figure S13). A third of the two populations were incubated separately in a static manner as a growth control. Another third was mixed together and incubated statically and the last third was mixed and incubated dynamically. The droplets were images after five hours of incubation with brightfield and fluorescence microscopy. For both mixed incubation conditions, less growth could be seen in the control droplets (Figure S13b). Due to the similar level of interdroplet transfer and due to the higher simplicity of the static incubation method, static incubation was done for all other experiments described in this work.


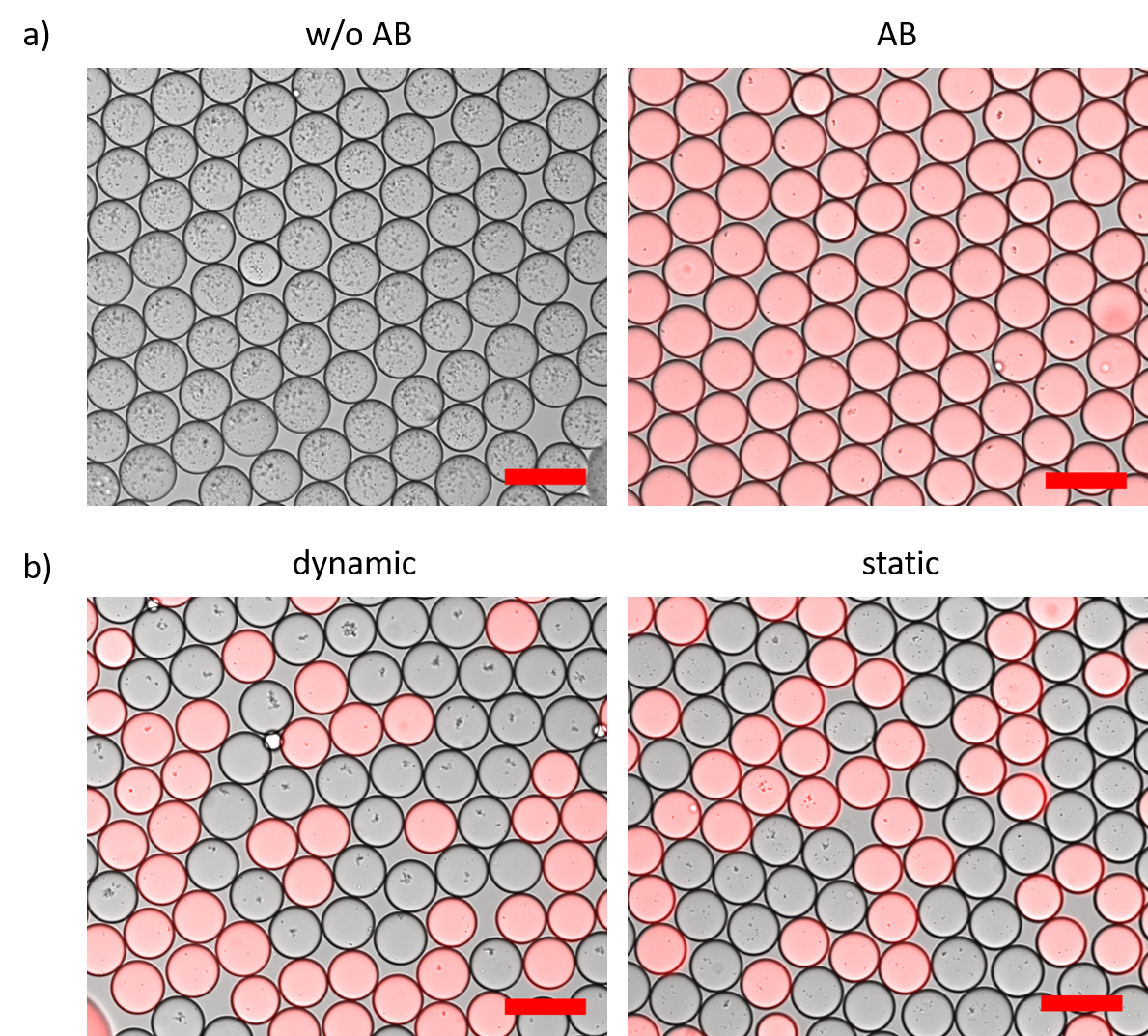


**Figure S13** Effect of incubation strategy on interdroplet transfer of 1 µg/mL ERY. a) Two droplet populations (without and with ERY + red fluorescence dye) were generated and incubated separately for 5 h as control. b) A portion of the droplet populations were mixed after generation and incubated either dynamically as described in Mahler et al.^[7]^ or statically in a microreaction tube with some oil and surfactant. The scalebars represent a length of 100 µm. Images were taken with 20 x magnification.

**4.3 Impact of various oils and surfactants on interdroplet transfer**

Different oil and surfactant combinations were tested to ideally find one that inhibits visible antibiotic leakage from one droplet to another through growth inhibition. For this, three additional oil/surfactant combinations were tested: ‘droplet generation oil for probes’ (contains a predefined concentration of surfactant by the manufacturer, Biorad) and Novec^TM^ HFE7500 perfluorinated oil with either 2 % dSURF (Fluigent), 0.5 % Pico-Surf (sphereFluidics), or 1 % Fluoro-Phase (Dolomite). Otherwise, the experimental design was the same as described in the other interdroplet transfer experiments with static droplet incubation. ERY was used again.

Unfortunately, growth was partially inhibited in all tested conditions (Figure S14). However, it seems like ERY could travel more easily into other droplets in some conditions than in others. There was very little cell growth in droplets with Biorad oil + surfactant, Novec oil + 2 % dSURF and Novec oil + 0.5 % Fluoro-Phase. In droplets with Novec oil + 0.5 % Pico-Surf, cell growth was also partially inhibited but more growth was visible as in the other three conditions. This could be due to a lower antibiotic concentration that leaked from antibiotic containing to non-antibiotic containing droplets. When comparing growth in Pico-Surf droplets to Fluoro-Surf (the original surfactant used) droplets incubated with droplets containing ERY, a similar inhibition could be seen. Therefore, we decided to stay with 0.5 % Fluoro-Surf droplets. However, as a conclusion, the incubation of different antibiotic conditions together is not possible. An alternative approach of generating and incubating the conditions separately and only mixing them just before fluorescence detection and ARS image acquisition was taken for AST. Future work is required to develop a strategy to avoid interdroplet transfer of antibiotics for fully integrated and simultaneous incubation.


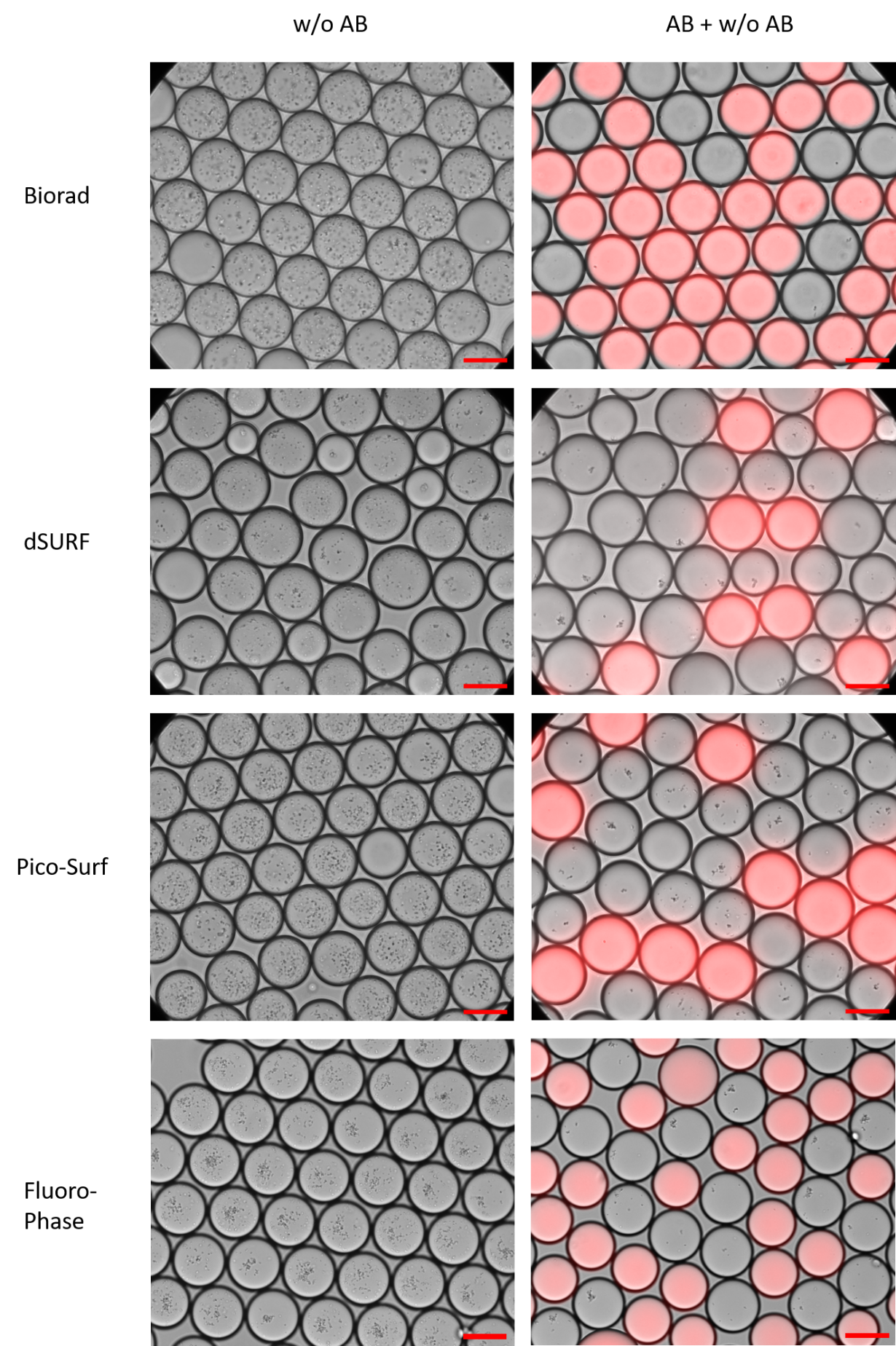


**Figure S14** Impact of different oils and surfactants on interdroplet transfer of 1 µg/mL ERY. Biorad: ‘droplet generation oil for probes’ (Biorad, contains already a surfactant) was used. dSURF: Novec^TM^ HFE7500 perfluorinated oil with 2 % dSURF (Fluigent) as surfactant. Pico-Surf: Novec^TM^ HFE7500 perfluorinated oil with 0.5 % Pico-Surf (sphereFluidics) as surfactant. Fluoro-Phase: Novec^TM^ HFE7500 perfluorinated oil with 1 % Fluoro-Phase (Dolomite) as surfactant. For all oil and surfactant combinations, an inhibition of growth in the non-antibiotic containing droplets when incubated together is visible. The scalebars represent a length of 50 µm.

# **AST results of all experiments**

The following graphs show the detailed results of the AST tests of four different *S. aureus* strains against five different antibiotics over time from 0 to 5 h after inoculation. Stripplots for all experiments represent the individual data points (Figure S15, S17, S19, S21), where each point represents the predicted value from an ARS image. The diamond shaped points represent the mean value of each condition. For the boxplot presentation of the same data (Figure S16, S18, S20, S22), the expected fraction of empty droplets for each antibiotic condition, as well as for the "w/o ab" population, was excluded. The fraction of empty droplets in the "w/o ab" condition was calculated using the maximum predicted value of the 99th percentile of ARS images classified as "empty." This value was employed as a threshold to estimate the percentage of droplets without cells in the "w/o ab" and antibiotic conditions. For the "w/o ab" condition, all predicted values below this threshold were removed for visualization. For the antibiotic conditions, if the percentage of droplets below the threshold was smaller than or equal to that in the "w/o ab" condition, all values below the threshold were also removed. However, if the percentage exceeded that of the "w/o ab" condition, only the equivalent percentage of values corresponding to the "w/o ab" condition below the threshold was removed. This approach enables a clear visual evaluation of the number of droplets containing cells that are susceptible to the tested antibiotic, without interference from empty droplets, which could otherwise obscure the interpretation of the data.


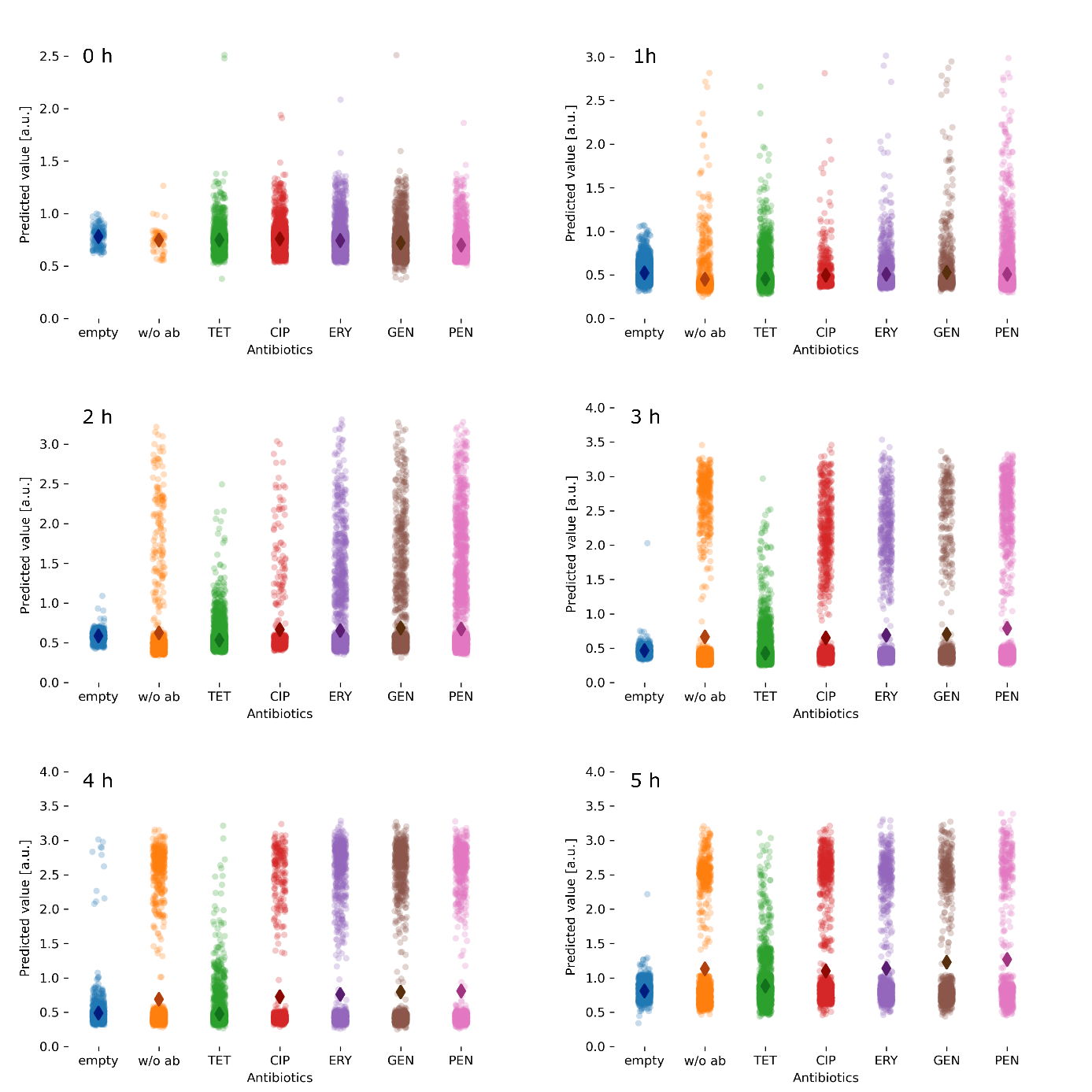


**Figure S15** AST results of S. aureus strain 1. All data is displayed.


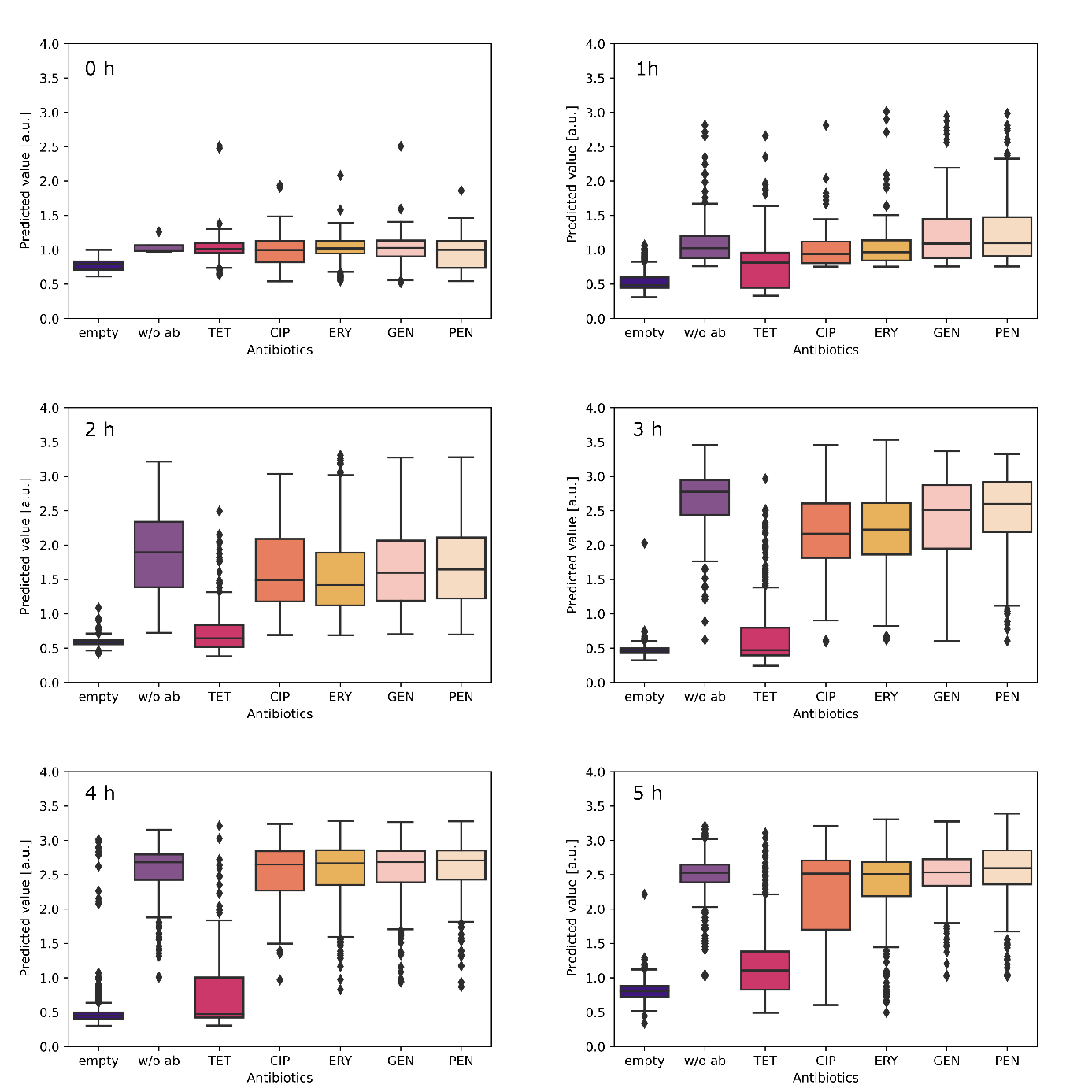


**Figure S16** AST results of S. aureus strain 1. Expected fraction of empty droplets was removed (see explanation above).


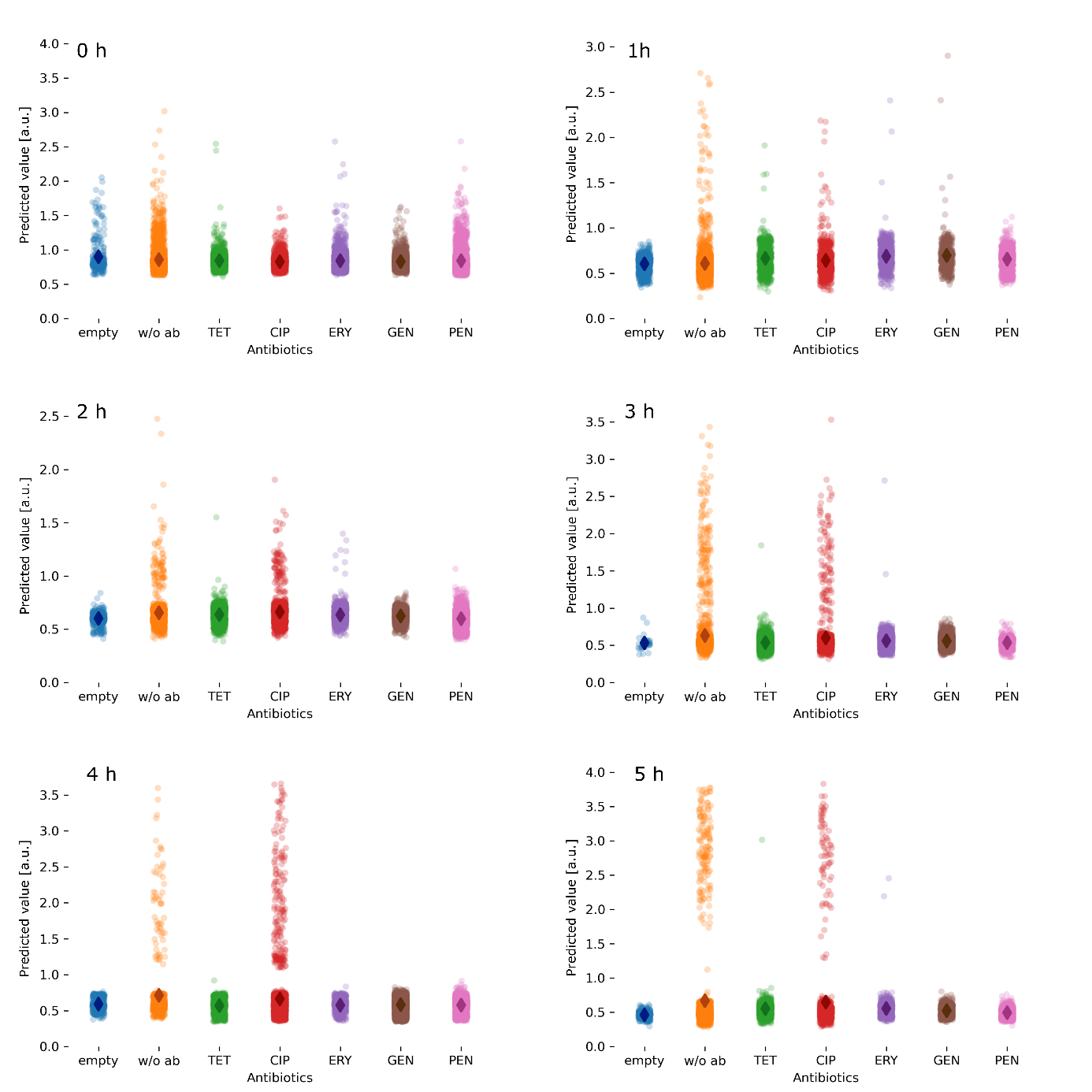


**Figure S17** AST results of S. aureus strain 2. All data is displayed.


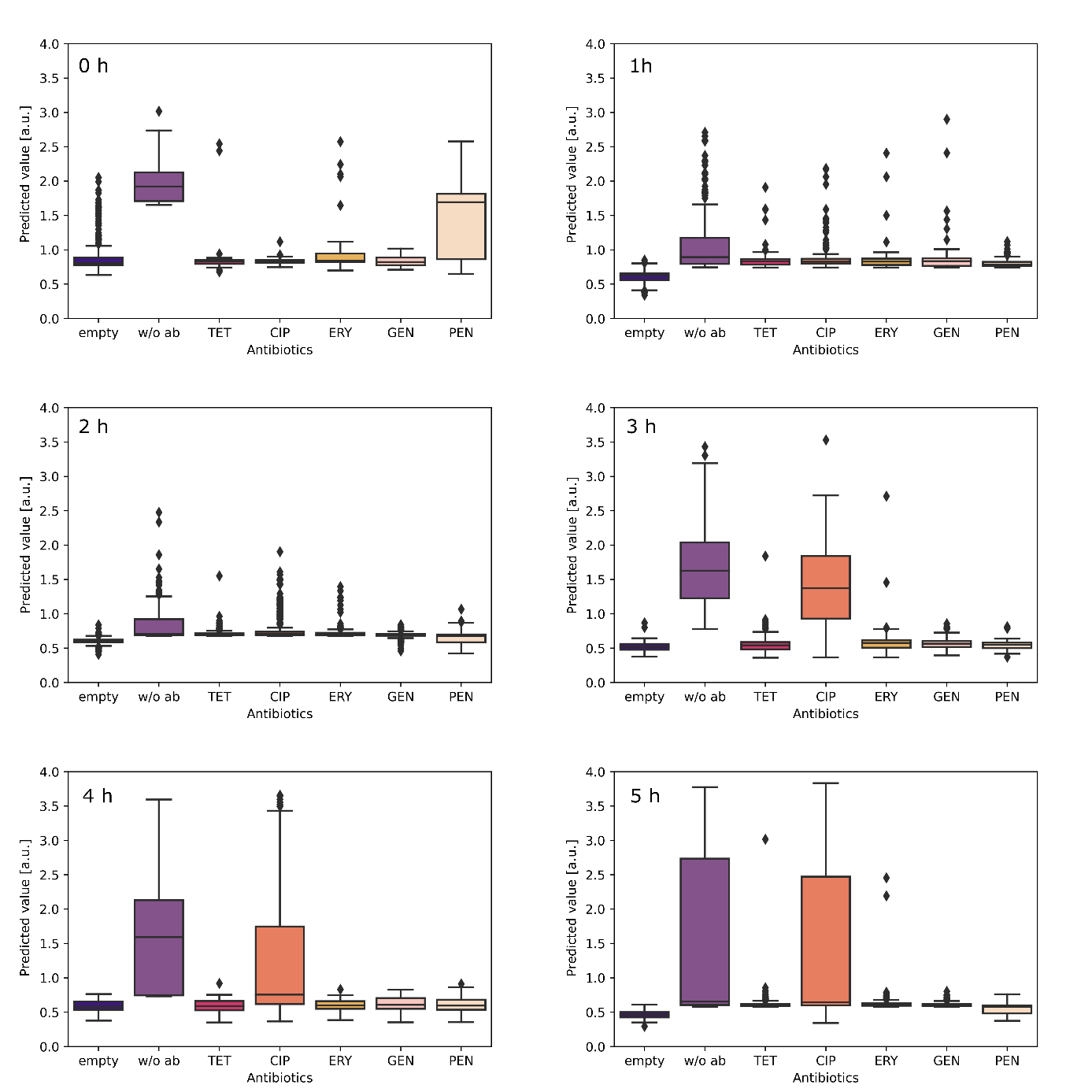


**Figure S18** AST results of S. aureus strain 2. Expected fraction of empty droplets was removed (see explanation above).


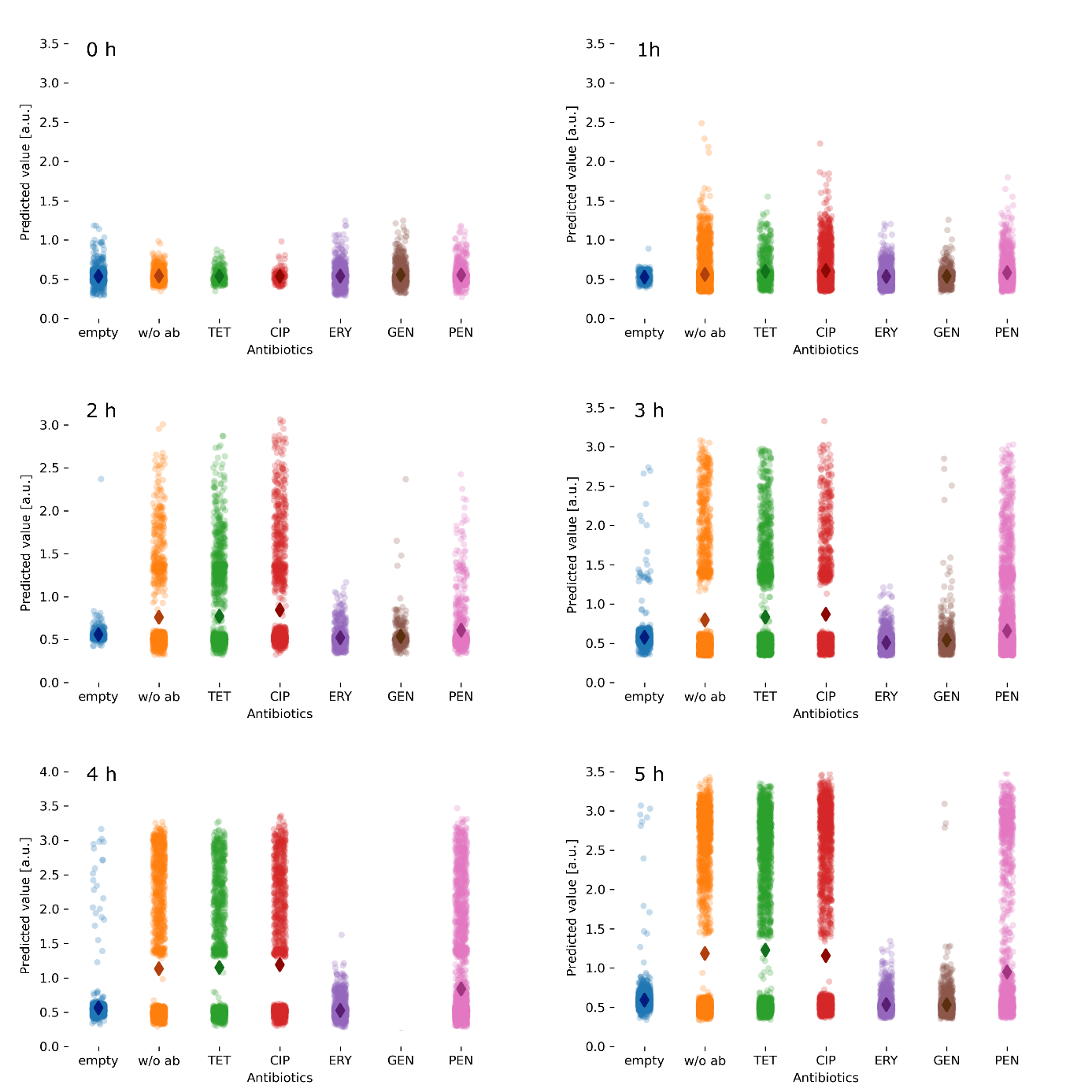


**Figure S19** AST results of S. aureus strain 3. All data is displayed.


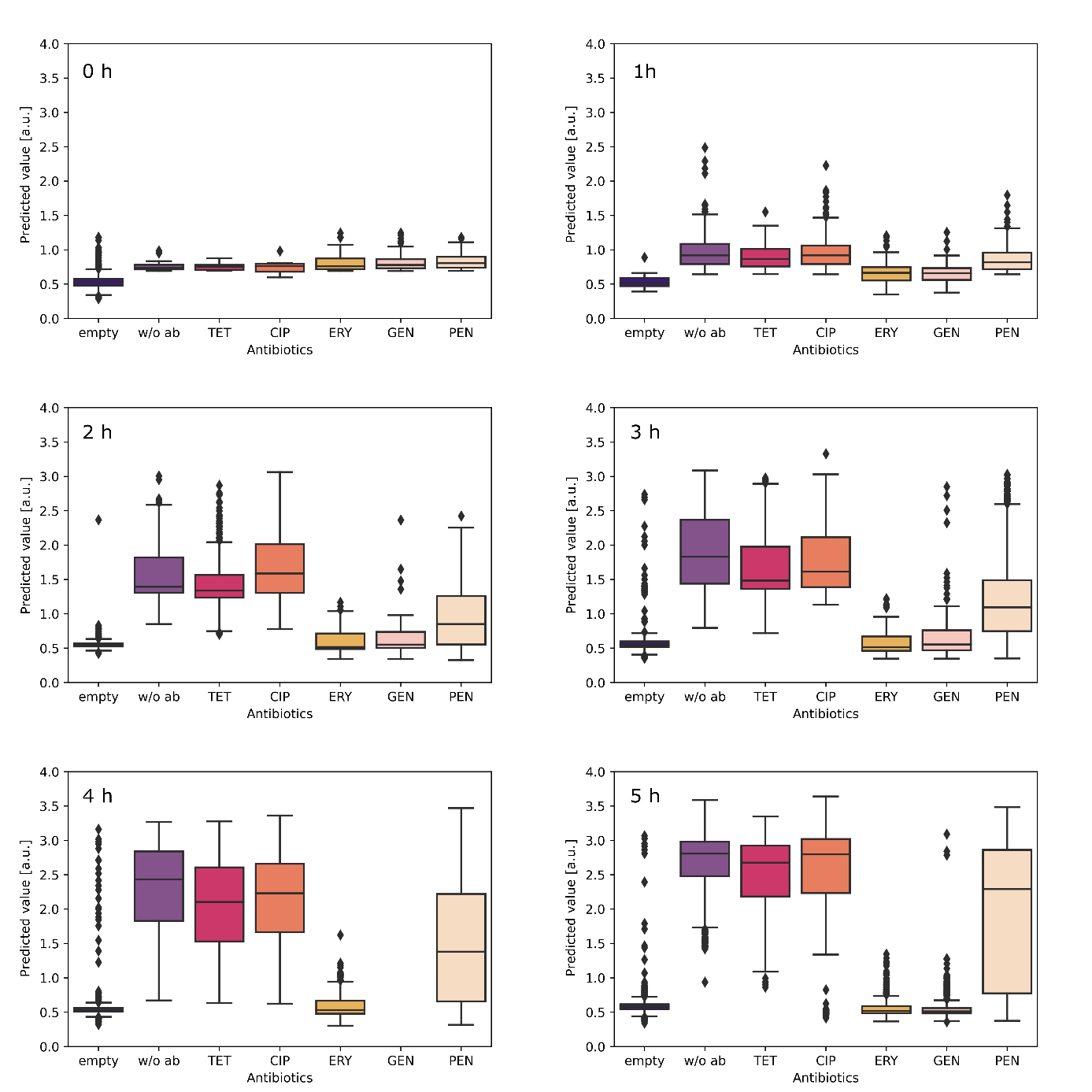


**Figure S20** AST results of S. aureus strain 3. Expected fraction of empty droplets was removed (see explanation above).


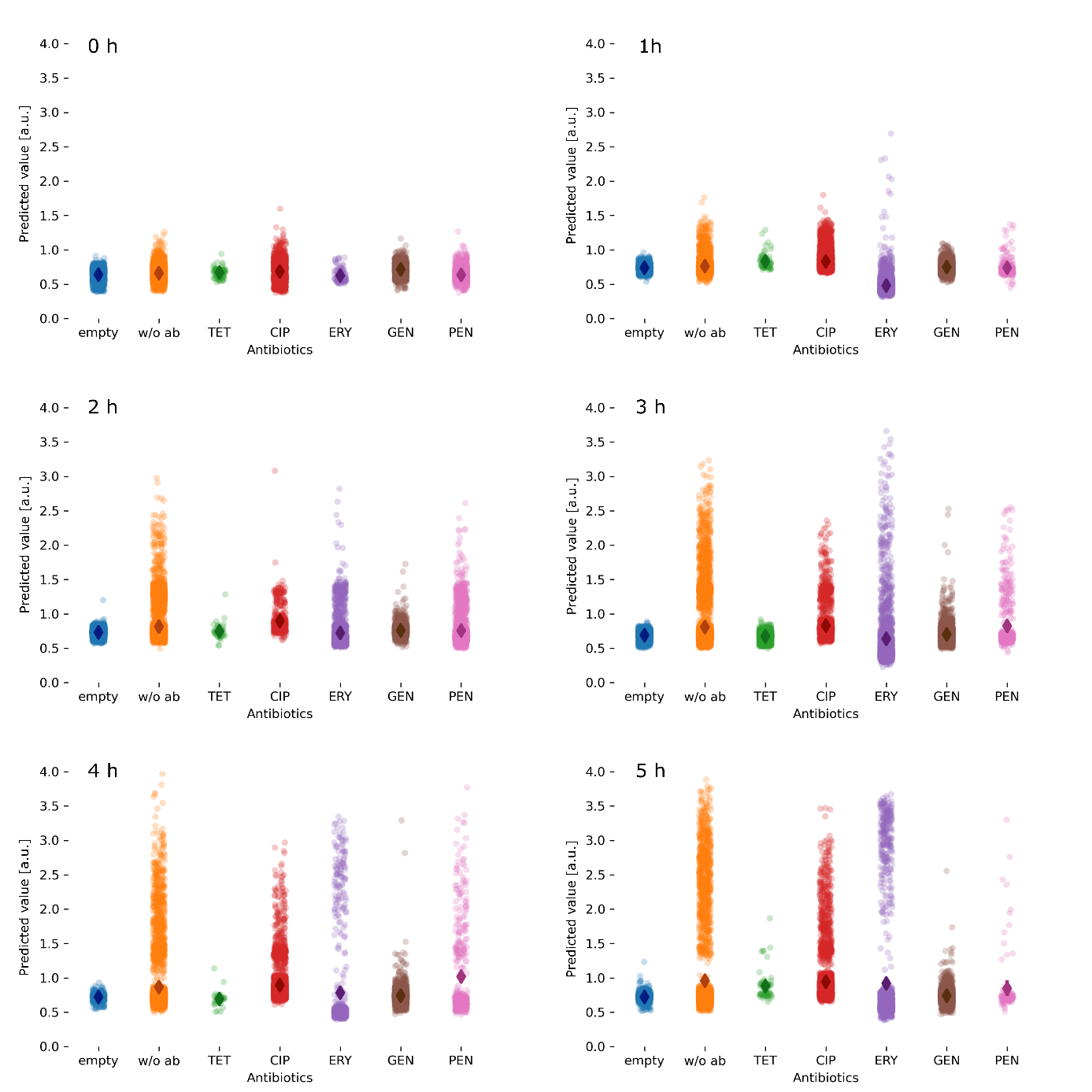


**Figure S21** AST results of S. aureus strain 4. All data is displayed.


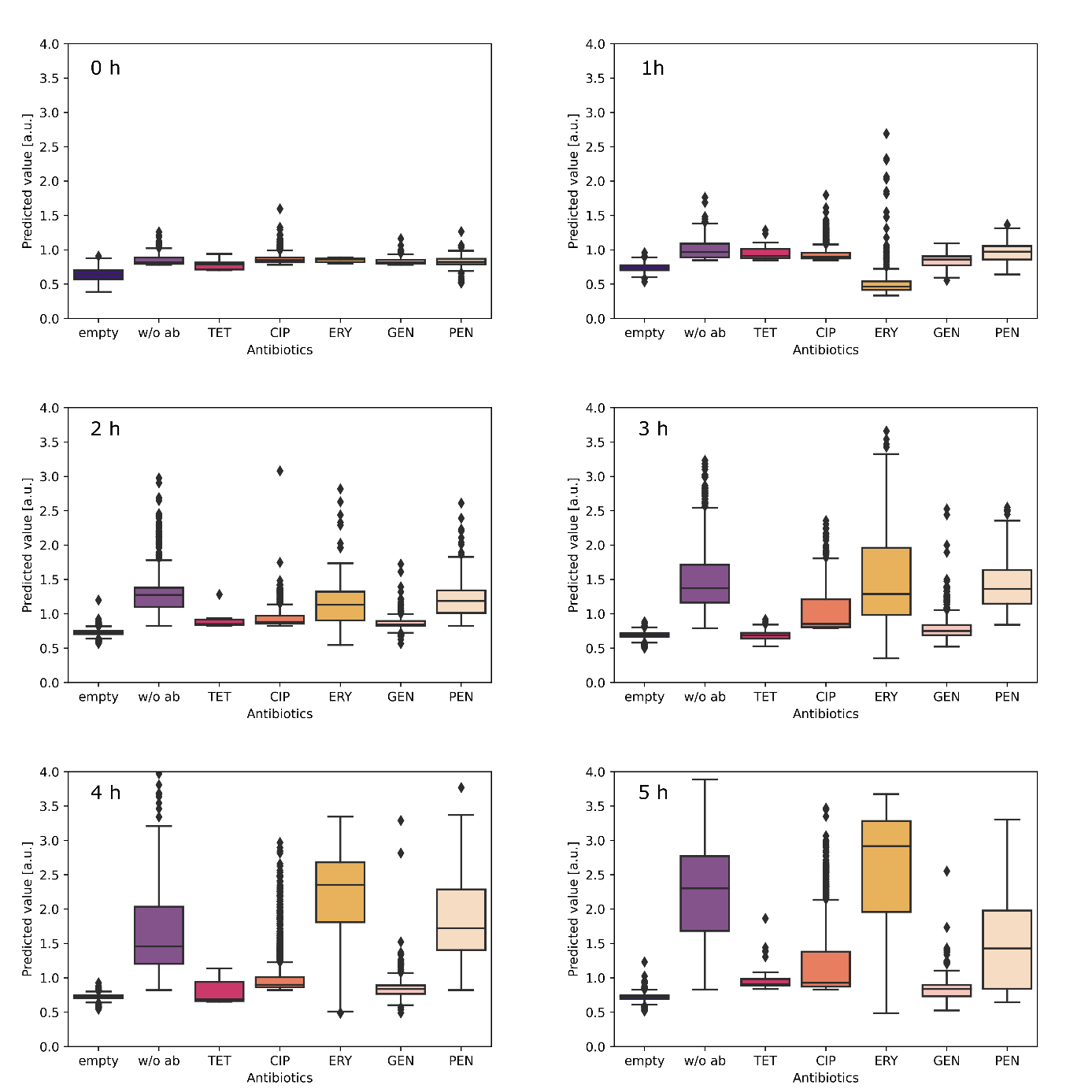


**Figure S22** AST results of S. aureus strain 4. Expected fraction of empty droplets was removed (see explanation above).

# **Disc diffusion results**

**Table S1** Measured inhibition zone diameters of various S. aureus strains when incubated with discs containing different antibiotics. The concentration and brand of antibiotic discs are mentioned in the parenthesis.

| *S. aureus* strain | Inhibition zone diameter [mm] | | | | |
| --- | --- | --- | --- | --- | --- |
|  | TET (30 µg, Difco, Order number: 6223) | CIP (5 µg, BD, Order number: 254724) | ERY (15 µg, Bio-rad, Order number: 66448) | GEN (10 µg, Difco, Order number: 6423) | PEN (1 unit, Oxoid, Order number: CT0152B) |
| 1 | 28 | 0 | 0 | 0 | 0 |
| 2 | 25 | 31 | 30 | 23 | 33 |
| 3 | 14 | 26 | 31 | 18 | 8 |
| 4 | 27 | 25 | 15 | 20 | 9 |

Abbreviations: TET: Tetracycline, CIP: Ciprofloxacin, ERY: Erythromycin, GEN: Gentamicin, PEN: Penicillin

# **References**

[1] M. Tovar, S. Hengoju, T. Weber, L. Mahler, M. Choudhary, T. Becker, M. Roth, *Anal. Chem.* **2019**, *91*, 3055.

[2] M. Tan, Q. Le, *Int. Conf. Mach. Learn.* **2019**, *PMLR 97*, 6105.

[3] M. Tan, Q. Le, *Int. Conf. Mach. Learn.* **2021**, *PMLR 139*, 10096.

[4] T. Ridnik, E. Ben-Baruch, A. Noy, L. Zelnik-Manor, in *35th Conf. Neural Inf. Process. Syst.*, **2021**.

[5] A. F. Agarap, *Deep Learning Using Rectified Linear Units (ReLU)*, **2018**.

[6] D. P. Kingma, J. Ba, in *3rd Int. Conf. Learn. Represent.*, **2014**.

[7] L. Mahler, M. Tovar, T. Weber, S. Brandes, M. M. Rudolph, J. Ehgartner, T. Mayr, M. T. Figge, M. Roth, E. Zang, *RSC Adv.* **2015**, *5*, 101871.
